# The role of social support on physical activity behaviour in adolescent girls: a systematic review and meta-analysis

**DOI:** 10.1186/s12966-016-0405-7

**Published:** 2016-07-07

**Authors:** Yvonne Laird, Samantha Fawkner, Paul Kelly, Lily McNamee, Ailsa Niven

**Affiliations:** Physical Activity for Health Research Centre (PAHRC), University of Edinburgh, St Leonard’s Land, Edinburgh, EH8 8AQ UK; School of Clinical Sciences, University of Edinburgh, Division of Psychiatry, Royal Edinburgh Hospital, Edinburgh, EH10 5HF UK

**Keywords:** Physical activity, Social support, Adolescent girls, Systematic review

## Abstract

**Abstract:**

Adolescent girls have been targeted as a priority group for promoting physical activity levels however it is unclear how this can be achieved. There is some evidence to suggest that social support could impact the physical activity levels of adolescent girls, although the relationship is complex and not well understood. We aimed to systematically review and meta-analyse the relationship between social support and physical activity in adolescent girls, exploring how different types and providers of social support might influence the relationship. Articles were identified through a systematic search of the literature using 14 electronic databases, personal resources, grey literature, and reference lists of included studies and previous reviews. Search terms representing social support, physical activity and adolescent girls were identified and used in various combinations to form a search strategy which was adapted for different databases. Cross-sectional or longitudinal articles published in English that reported an association between social support and physical activity in adolescent girls between the ages of 10 to 19 years were included. Studies that focused only on clinical or overweight populations were excluded. Data extraction was carried out by one reviewer using an electronic extraction form. A random 25 % of included articles were selected for data extraction by a second reviewer to check fidelity. Risk of bias was assessed using a custom tool informed by the Critical Appraisal Skills Programme Cohort Study Checklist in conjunction with data extraction. Cross-sectional results were meta-analysed and longitudinal results were presented narratively. Small but significant associations between all available providers of total social support (except teachers) and physical activity were found (*r* = .14-.24). Small but significant associations were also identified for emotional, instrumental and modelling support for some providers of support (*r* = .10-.21). Longitudinal research supported the cross-sectional analyses. Many of the meta-analysis results suggested high heterogeneity and there was some evidence of publication bias, therefore, the meta-analysis results should be interpreted with caution. In conclusion, the meta-analysis results suggest that social support is not a strong predictor of physical activity in adolescent girls though parents and friends may have a role in enhancing PA.

**Trial registration:**

PROSPERO 2014:CRD42014006738

**Electronic supplementary material:**

The online version of this article (doi:10.1186/s12966-016-0405-7) contains supplementary material, which is available to authorized users.

## Background

The health benefits of regular physical activity (PA) are well documented [1], yet there are concerns about the low levels of PA in adolescents. It has been estimated that 80 % of adolescents worldwide fail to achieve PA guidelines [2], with adolescent girls consistently identified as less active than boys [2–4]. There is also evidence to suggest that activity levels during adolescence may track into adulthood [5]. As a result, interventions have been developed that aim to promote PA in adolescent girls, although these have had limited effect (6–8). Recent evidence also suggests that there are gender differences in correlates of physical activity in adolescents [9]. Therefore, understanding the correlates and determinants of PA specifically in adolescent girls is essential to inform the development of current and future interventions for this population [10].

A growing body of evidence focusing on correlates and determinants of PA in adolescent girls exists, and subsequently, research has been summarised by systematic reviews [11–14]. Consistent with a socio-ecological approach [15], these reviews have identified categories of PA correlates including personal, psychological, environmental and social correlates. Social support in particular has consistently emerged as positively related to PA in adolescent girls. Social support describes resources provided from interactions with significant others that can influence behaviour [16, 17]. These resources can be emotional (e.g. encouragement, praise), instrumental (e.g. equipment, financial), or informational support (e.g. advice, instruction) and they can be provided by various individuals (providers) within one’s social network (e.g. friends, family, teachers) [18, 19]. Within the PA literature, modelling (e.g. associations between activity levels of provider and child) and co-participation (e.g. performing physical activities together) have also been considered forms of social support [20, 21]. Table 1 outlines these different types of support. In social support measurement studies, social support typically refers to a composite score of one or more of these types of sub-types of support.

**Table 1:** Types of social support for physical activity

Type of support	Sub-types of support/description
Emotional support	Providing child with encouragement for physical activities; encouraging child to be active; talking to child about physical activities; praise; watching child perform physical activities
Instrumental support (logistic support)	Financial support; providing transport to physical activities; providing equipment for child to be physically active (e.g. bicycle)
Informational support	Feedback on physical activities; providing instruction or advice to be physically active
Co-participation	Performing activities with child (e.g. going for walks together)
Modelling	Provider ‘models’ PA and child modifies their behaviour/associations between activity levels of provider and child
Total social support	A composite score of social support, typically refers to one or more of the above sub-types of support

Numerous studies have focused on social support for PA in children and adolescents and these have also been systematically reviewed [14, 20, 22–28]. Most of these reviews have focused on parental influences with results suggesting positive significant associations between parent support and child and adolescent PA [20, 22–24, 26, 27]. Despite the lower prevalence of PA in adolescent girls, none of these reviews focused specifically on this group. Only two reviews considered gender and they found no significant differences between boys and girls, however, analyses only considered overall support [20, 27] and modelling [20] and were presented for both children and adolescents. There is some evidence to suggest the relationship between social support and PA might vary by age [23] and gender [29], therefore, considering these variables separately may better inform PA intervention development for adolescent girls.

Pugliese and Tinsley [27] and, later, Yao and Rhodes [20] conducted the two meta-analyses in the area. They both identified small to medium significant associations between parent support and youth PA (*r* = .17 and *r* = .38 respectively) and small but significant associations between parent modelling and youth PA (*r* = .13 and *r* = .16 respectively). Yao and Rhodes [20] also found that parental encouragement and co-participation were most strongly related to youth PA compared with praise, watching and logistic support, perhaps suggesting that different types of social support may influence PA differently. Neither meta-analysis considered all providers of social support. This limits our understanding about the relative importance of different types and providers of social support for adolescent girls’ PA.

Yao and Rhodes [20] also considered how other variables moderated the effect sizes of the relationship. Specifically, they found that PA measure (e.g. objective/subjective) moderated one of the effect sizes, with subjective tools showing larger effects. Study quality, geographical location and age were also assessed but did not significantly moderate effect sizes. It is also possible that other factors not investigated could have influenced reported effect sizes. For example, there is some evidence to suggest that associations may vary according to type of PA (e.g. sports, active travel) [24]. Measurement of social support may also moderate effect sizes, as inconsistent methods of measuring social support and the use of non-validated scales has previously been highlighted as problematic in the literature [30]. Similarly, it is possible that effect sizes differ based on whether social support is reported by the child (perceived support) or reported by the provider (received support) For example, parents may believe that they are supporting their child to be active (received support) but if the child does not feel supported (perceived support) then the association with PA is likely to differ between perceived and received support.

To date, the only available evidence that has considered all providers and all types of social support on PA in young people adopted a semi-quantitative and narrative approach [22]. Whilst the findings from this review suggested that support from both parents and friends is positively associated with PA in adolescents, no comparison in effects sizes between providers and types of support was possible. Performing meta-analysis would allow us to compare effect sizes and establish if some types and providers of support are more strongly associated with PA in adolescent girls than others. Understanding these relationships more comprehensively could inform PA intervention design for adolescent girls. In particular, this analysis could inform the providers and types of social support that should be targeted in PA interventions for adolescent girls.

Therefore, the first aim of this study was to comprehensively map the literature to demonstrate the numbers of associations reported for different combinations of types and providers of social support. Secondly, where there was enough available evidence, we aimed to perform meta-analyses on effect size data for different providers and types of social support for adolescent girls’ PA. Finally, we aimed to carry out moderator analyses on effect sizes for age, geographical location, social support measurement bias (e.g. high risk, low risk), who reported the social support (e.g. perceived or received support), PA measure (objective or subjective) and type of PA (e.g. active travel, sports).

## Methods

This study followed the procedures for systematic reviews and meta-analysis outlined in the PRISMA statement [31]. A protocol for this review was prepared and registered with PROSPERO [32].

### Search strategy

Literature published until January 2015 were synthesised and reviewed. The following electronic databases were searched to identify studies for inclusion: MEDLINE, PsychINFO, EMBASE, CABabstracts, Global Health, Allied and Complementary Medicine, SPORTDiscus, ERIC, CinAHL, Science Citation Index, Social Science Citation Index, the Cochrane library, Dissertations and Theses A&I and the International Bibliography of the Social Sciences. Additional articles were located using the reference lists of included articles and previously published reviews. Personal resources including the authors’ own EndNote libraries and book chapters were consulted. Search terms included a combination of free text terms and subject headings relating to the target population, social support, and PA (see Table 2). The search strategy was adapted for each database and searches were logged and recorded. Pilot searches were conducted to improve the sensitivity and specificity of the final search strategies.

**Table 2:** Systematic review search terms

Target population	Social support	Physical activity
Adolescen*	Social support	Sport*
Young people	(Family or peer or friend* or school) adj2 (support or encourage* or help or assist*)	Physical activit*
		Physical fitness
Youth	(emotion* or instruction* or information* or psychosocial) adj2 (support or encourage* or help or assist*)	Exercis*
Girl*		
Female*		
Teen*		
School age*		

* Search term truncated

### Eligibility criteria

Peer reviewed publications or doctoral theses published until January 2015 were included. No limitation was placed on start date. Studies were eligible for inclusion if: (1) data for adolescent girls between the ages of 10 to 19 years, or a mean age within this range, were reported (based on the World Health Organizations [33] definition of adolescence) (2) they included a measure of social support as an independent variable, (3) they included a measure of adolescent’s PA as a dependent variable, and (4) they reported an association between PA and social support (e.g. quantitative studies reporting cross-sectional or longitudinal associations). Studies were excluded if (1) they focused only on clinical or overweight populations, (2) only a health related fitness measure was reported, or (3) they were not published in English.

### Screening

Two reviewers independently screened search results against the inclusion and exclusion criteria. This was carried out in two stages. The initial stage involved screening titles and abstracts only, and full articles were located where titles and abstracts were identified as meeting the inclusion criteria. Any disagreements were discussed and resolved during a meeting with a third reviewer.

### Data extraction and risk of bias assessment

Data from the included articles were extracted onto an electronic form, which was designed and piloted for this review. The extracted data included: general study information; participant characteristics; outcome characteristics for PA and social support; methods of analysis; and results. In conjunction with data extraction, included studies were assessed for risk of bias. The Critical Appraisal Skills Programme for cohort studies tool (CASP; www.casp-uk.net), a checklist based on a tool used previously in the PA literature [34], was used to guide risk of bias assessment. Four categories relating to study sampling and instrument validation were identified that might pose a risk of bias to the type of studies likely to be included in the review, including: selection bias, PA measurement bias, social support measurement bias, and confounding variables. Each category within each study was then assigned as having a ‘low’, ‘high’ or ‘unclear’ risk of bias using an 8-item checklist of pre-determined assessment thresholds (see Additional file 1). The risk of bias assessment was not used to exclude or weight studies within the review. Data extraction and risk of bias assessment was completed by one author. To estimate accuracy, a second reviewer carried out data extraction and risk of bias assessment on a random 25 % of the included studies. Following this, any disagreements were resolved during a meeting with a third reviewer. The inter-rater reliability for the two reviewers was found to be Kappa = 0.62, suggesting a good level of agreement between the two reviewers [35]. Therefore, the data extraction and risk of bias accuracy of one reviewer was deemed to be acceptable.

### Effect size calculation

Random effects meta-analyses were performed using Comprehensive Meta-Analysis Software Version 3.0 [36] to estimate pooled associations between provider and types of social support and PA in adolescent girls. Adjusted (where available) and non-adjusted (if adjusted not reported) standardised effect size metrics or odds ratios were entered into Comprehensive Meta-Analysis (e.g. bivariate correlations, standardised regression coefficients). In cases where standardised effect sizes were not available, if available, *p*-values and sample sizes were entered into Comprehensive Meta-Analysis and the effect size was back computed. If only non-standardised effect sizes were available, studies were not meta-analysed. Where a study reported more than one effect size for one association (e.g. parent support on PA) an overall effect size was included in the meta-analysis. If this was not available (for example, effect sizes were separated by ages and not reported overall) then more than one effect size for a study was entered into the meta-analysis and highlighted in the results table. In cases where multiple forms of PA were reported then moderate-to-vigorous PA (MVPA), or the closest form of activity to MVPA, was included in the meta-analysis. Effect sizes were converted to the Fisher’s z scale, and all analyses were performed using the transformed values before being converted back to correlations to present the results. Pearson’s *r* was selected as the effect size metric to report the results and interpretation of the results were based on Cohen’s criteria for small (>0.10), moderate (>0.30) and large (>0.50) effect sizes [37]. Meta-analyses were performed for different types and providers of social support, providing at least 3 studies reported results on the combination of provider and type of support. Previous reviews informed the selection of six possible moderators of effect sizes (see Additional file 2) [20, 24]. Effect sizes were assessed for these proposed moderators by meta-regression including age, geographical location, social support measurement bias, who reported the social support (e.g. perceived or received support), PA measure (e.g. subjective or objective) and PA type (e.g. MVPA, sport) when at least six studies were included in the meta-analysis. Who reported social support was not assessed as a moderator for modelling. This was because modelling was measured by three mechanisms: self-report by provider, child reported modelling, and using objective measures. Use of objective measures is assessed as a separate moderator, and it was not possible to complete the analyses only for the subjective measures.

### Longitudinal studies

Longitudinal studies were not included in the meta-analysis and were presented narratively. This was deemed the most appropriate way to represent the longitudinal data due to the varied analyses performed. For example, the predictive effect of baseline social support on future PA is not directly comparable to change in social support and PA over time. It was, therefore, deemed inappropriate to statistically pool these findings.

## Results

**Fig. 1:**
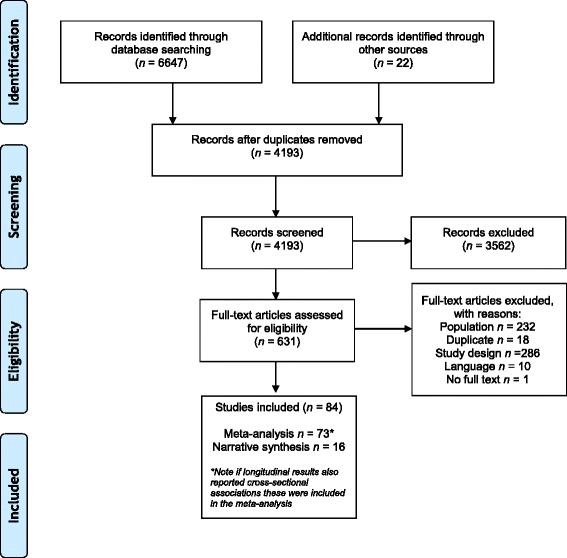
Search flow diagram

**Fig. 2:**
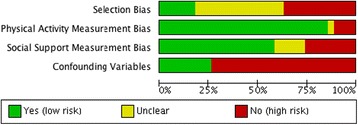
Risk of bias of included studies

A total of 6647 records were identified from electronic and manual searches, of which 84 met the inclusion criteria (see Fig. 1). Of these, data from 73 studies were included in the meta-analysis and data from 16 longitudinal studies were included in the narrative synthesis. Six cross-sectional studies were not included in the meta-analysis because there were not enough data to perform a meta-analysis [38–41] or because data could not be meta-analysed [42, 43]. Included studies were published between 1986 and 2014. The majority of studies were conducted in the USA (55 %). Other studies were conducted in Europe (15 %), Australia (12 %), Asia (8 %), Canada (7 %), and South America (2 %). Most studies were cross-sectional in design (81 %), measured PA subjectively (71 %), and included participants aged between 13 and 15 years (52 %) (see Additional file 3). Included studies were assessed for risk of bias (see Fig. 2). As shown in the figure, most studies were of high risk of selection bias or did not report the relevant information on study selection. The majority of studies (75 %) did not control for all the proposed confounding variables in the risk of bias assessment and just over half of the included studies used a validated tool to measure social support (see Fig. 2).

Table 3 provides an overview of the associations reported by the included studies, representing the combinations of associations available for 21 different providers and 14 types of social support for adolescent girls. Associations were predominantly reported for total social support by all providers, parents, family and friends on adolescent girls’ PA. Total social support refers to an overall measure of social support for PA, this could include various sub-types of social support. Similarly, ‘all providers’ refers to studies that have not specified a provider of social support or have combined providers (e.g. parents, friends) using a composite score for social support. Associations were also commonly reported for modelling, particularly for parents, mothers, fathers and friends. Associations for other types of support such as emotional, instrumental or informational support were rarely reported and some providers of support were not investigated by many researchers (such as teachers, coaches and siblings) (see Table 3).

**Table 3:** Number of social support associations with adolescent girls’ physical activity reported by provider and type

Provider	Total support	Emotional support^a^					Instrumental support^b^				Modelling	Co-participation	Guiding	Informational
		Em	En	Pr	Ta	W	In	Tr	F	L				
All providers	12										1	1		
Parents	14		7		1		2	3	1		14	4	1	
Family	33	1	1	1			1					1		1
Mother	5		4			1	1			3	11			
Father	5		3				1			2	9	1		
Friend	35		1								10			
Teacher	6		1				1				1			
Sibling	2													
Brother											1			
Sister											1			
Best friend											1			
Boy peers	2		1								1			
Female peers	1		1								1			
Adult											1			
Coach	1										1			
Classmate											1			
Boy/girlfriend											1			
Primary caregiver											1			
First nominated friend											1			
Second nominated friend											1			
Third nominated friend											1			

^a^ *Em* Emotional, *En* Encouragement, *Pr* Praise, *Ta* Talking, *W* Watching

^b^ *In* Instrumental, *Tr* Transport, *F* Financial, *L* Logistic

### Total social support

The relationships between different providers of total social support and PA in adolescent girls were estimated by random effects meta-analysis (see Table 4). Small but significant associations were identified for every available provider of social support except teachers on adolescent girls PA (*r* = 0.14-0.24). However, there was considerable heterogeneity for most of the associations suggested by the significant Q-values and the high *I*^*2*^ statistics.

**Table 4:** Associations from meta-analysis of all providers and sub domains of providers of total social support with physical activity outcomes

	Reference numbers of included studies	Effect size statistics					Heterogeneity statistics				Publication bias
		*k*	*r*	SE	S^2^	95 % CI	*Z*	*Q*	τ^2^	*I* ^*2*^	Fail safe *N*
All providers	[40, 49, 57, 58, 65, 70–76]	12	0.237	0.012	0.000	0.150, 0.321	5.21^***^	76.062^***^	0.017	85.54	555
Parents	[48, 77–89]	14	0.192	0.012	0.000	0.108, 0.273	4.50^***^	116.43^***^	0.020	88.83	513
Family	[50, 52, 73, 76, 90–106]^a^	32	0.136	0.009	0.000	0.081, 0.191	4.79^***^	420.96^***^	0.023	92.40	1815
Mother	[59, 70, 107]	3	0.223	0.004	0.000	0.163, 0.280	7.20^***^	1.974	0.000	0.000	31
Father	[59, 70, 107]	3	0.161	0.003	0.000	0.101, 0.219	5.25^***^	1.119	0.000	0.000	17
Friend	[52, 55, 59, 73, 78, 79, 86, 88, 90, 93–95, 97, 98, 100, 102–112]^a^	33	0.135	0.004	0.000	0.096, 0.173	6.75^***^	180.23^***^	0.009	82.24	1738
Teacher	[52, 104, 110]^a^	6	0.062	0.015	0.000	−0.051, 0.174	1.08	102.55^***^	0.019	95.12	3

*P < 0.05, ***P < 0.001

*k* = number of studies; *r* = effect size; SE = standard error; S^2^ = variance; 95 % CI = 95 % confidence interval; *Z* = test of null hypothesis; *Q* = total Q-value used to assess heterogeneity; τ^2^ = between study variance; *I* ^*2*^ = the percentage of total variance across studies not attributed to sampling error; Fail safe N = the number of additional studies (in which the effect was zero) that would be needed to increase the meta-analysis *P* value to above 0.5. ^a^ = More than one effect size included in the meta-analyses from the following studies [50, 91, 93, 99, 100]

Moderator analyses did not find any of the proposed moderators to be significant for total support from all providers, parents or friends (*p* > 0.05). For family support, PA type was a significant moderator of the association between family support and PA. Associations for sports participation (*r* = 0.44, 95 % CI 0.19-0.69) were significantly higher (*p* < 0.01) than MVPA (*r* = 0.04, 95 % CI −0.06-0.14), total PA (*r* = 0.10, 95 % CI −0.01-0.21), after school PA (*r* = 0.03, 95 % CI −0.16-0.22), and active travel (*r* = −0.14, 95 % CI −0.40-0.12). There were not enough studies included in the mother, father or teacher support meta-analyses to perform moderator analysis.

### Sub-domains of social support by provider

#### Emotional support

The most commonly reported form of emotional social support was encouragement, with only five other studies reporting additional types of emotional social support (talking *n* = 1, watching *n* = 1, praise *n* = 1, overall emotional support *n* = 1). Due to these low numbers for other forms of emotional support, and because different forms of emotional support may influence PA in different ways, we decided to perform analyses only on associations between encouragement and PA (see Table 5). Small but significant associations were identified for every provider of encouragement on adolescent girls PA (*r* = 0.10-0.21). However, there was significant heterogeneity for most of the associations except for father encouragement. Due to the low sample sizes in the meta-analyses, moderator analysis was only performed for parent encouragement and no significant moderators were identified (*p* > 0.05).

**Table 5:** Associations from meta-analysis of providers of sub-domains of support with physical activity outcomes

	Reference numbers of included studies	Effect size statistics					Heterogeneity statistics				Publication bias
		*k*	*r*	SE	S^2^	95 % CI	*Z*	*Q*	τ^2^	*I* ^*2*^	Fail safe *N*
Encouragement											
Parents	[45, 82, 113–117]	7	0.103	0.006	0.000	0.032, 0.173	2.841*	31.29***	0.007	80.824	108
Mother	[70, 115, 118–120]	5	0.194	0.015	0.000	0.111, 0.275	4.512***	8.222	0.004	51.349	53
Father	[70, 115, 120]	3	0.211	0.003	0.000	0.153, 0.266	7.075***	1.126	0.000	0.000	36
Instrumental support											
Parents	[45, 105, 115, 121, 122]^a^	6	0.169	0.002	0.000	0.131, 0.206	8.648***	5.545	0.000	9.822	107
Mother	[119, 123–125]	4	0.214	0.022	0.000	0.060, 0.359	2.703*	13.26*	0.019	77.37	26
Father	[119, 124, 125]	3	0.234	0.050	0.002	−0.011, 0.452	1.875	12.827^*^	0.040	84.41	13
Modelling											
Parents	[45, 52, 74, 82, 86, 87, 92, 96, 105, 113, 116, 117, 126, 127]	14	0.130	0.011	0.000	0.049, 0.209	3.154*	105.788***	0.019	87.711	214
Mother	[49, 53, 70, 78, 89, 105, 115, 124, 125, 128, 129]	11	0.079	0.012	0.000	−0.004, 0.160	1.874	104.625***	0.014	90.442	101
Father	[70, 78, 89, 105, 115, 124, 125, 128, 129]	9	0.144	0.011	0.000	0.054, 0.232	3.128*	54.458***	0.014	85.310	131
Friends	[51, 52, 78, 89, 104, 110, 114, 126, 127, 130]	10	0.161	0.013	0.000	0.074, 0.245	3.615***	191.764***	0.017	95.307	505
Co-participation											
Parents	[45, 105, 122, 126]	4	0.033	0.017	0.000	−0.102, 0.168	0.483	34.00	0.017	91.18	0

*P < 0.05, ***P < 0.001

*k* = number of studies; *r* = effect size; SE = standard error; S^2^ = variance; 95 % CI = 95 % confidence interval; *Z* = test of null hypothesis; *Q* = total Q-value used to assess heterogeneity; τ^2^ = between study variance; *I* ^*2*^ = the percentage of total variance across studies not attributed to sampling error; Fail safe N = the number of additional studies (in which the effect was zero) that would be needed to increase the meta-analysis *P* value to above 0.5. ^a^ = More than one effect size included in the meta-analysis for study: [122]

#### Instrumental support

Studies that provided associations between instrumental support and PA were less common; with providers including parents, mothers and fathers (see Table 5). Studies were included in the instrumental support meta-analyses if they reported on relationships between general instrumental support, transport, financial, or logistic support for PA. These types of instrumental support were combined to form a composite instrumental support effect size, due to the low numbers of individual instrumental support associations reported. Significant associations were identified for parents and mother instrumental support on adolescent girls’ PA (*r* = 0.17-0.21), but father instrumental support was not significant (*r* = 0.23).

Due to the low sample sizes, moderator analysis was only performed for parent instrumental support. As only six studies were available, separate models had to be conducted for each proposed moderator. This identified age and geographical location as significant moderators of parent instrumental support. Studies conducted in the USA had larger effect sizes (*r* = 0.20, 95 % CI 0.16-0.24) than those conducted in Australia (*r* = 0.09, 95 % CI 0.01—0.18). Effect sizes were significantly higher (*p* < 0.05) for girls aged 13 to 15 years (*r* = 0.20, 95 % CI 0.16-0.25) compared with younger girls aged 10 to 12 years (*r* = 0.09, 95 % CI 0.01-0.18).

#### Modelling and co-participation

Small but significant associations were identified for parents, father, and friend modelling on adolescent girls PA (*r* = 0.13-0.16) (see Table 5). No significant associations were found for modelling by mothers or family modelling on adolescent girls’ PA. However, there was significant heterogeneity in all of the associations. Few studies investigated associations between co-participation and adolescent girls PA. There were only enough studies reporting associations for parents, and parent co-participation was not found to be significantly related to adolescent girls PA (*r* = 0.03).

Moderator analyses was performed for parent, mother, father, and friend modelling. No significant moderators were identified for parent, mother, or friend modelling (*p* > 0.05). The relationship between father and adolescent PA was also moderated by how the girls’ PA was measured (*p* < 0.05). To demonstrate this, subjective measures showed higher effect sizes (*r* = 0.25, 95 % CI 0.04-0.46) compared with objective measures (*r* = −0.03, 95 % CI −0.28-0.22). There were not enough studies included in the parent co-participation meta-analysis to perform moderator analysis.

### Publication bias

Funnel plots (available from corresponding author) were inspected for evidence of publication bias, which suggested possible publication bias for friend modelling and PA. Fail-safe N analysis was subsequently conducted. This found that 505 additional studies in which the effect was zero would be needed for the overall effect to be statistically insignificant. This suggests a possible skewed effect size. However, subsequent trim and fill analysis did not suggest it was necessary to trim studies from the analysis, therefore, the effect size remained the same. For other analyses, fail-safe N suggested that few additional studies (<150) were needed for the overall effect to be statistically insignificant in many of the meta-analyses performed. This suggests a possible skewed effect size although this could be linked to low sample sizes in the meta-analyses.

### Longitudinal findings

Longitudinal associations between social support and PA in adolescent girls were investigated in 16 studies [44–59]. Different methodological approaches were used to assess these associations. This included assessments of baseline social support as a predictor of follow-up PA, repeated cross-sectional analyses, and assessment of changes in social support and PA over time. The following section provides an overview of the results of these analyses.

#### Total social support

As shown in Table 6, a total of 12 studies examined the relationship between total social support and adolescent girls’ PA longitudinally, of which 10 studies reported a positive association and two reported no association. Positive associations were identified for general providers (*n* = 1), parents (*n* = 2), family (*n* = 3) and friends (*n* = 4), whilst the two studies identifying no associations were for general providers.

**Table 6:** Longitudinal associations between social support and physical activity in adolescent girls

	Total support			Encouragement			Instrumental			Modelling			Co-participation		
	+	-	0	+	-	0	+	-	0	+	-	0	+	-	0
General providers	[58]		[53, 57]									[53]			
Parents	[47] [48]					[45]	[45]			[52]		[45]	[46]		[45]
Father										[44, 46]					
Mother				[44]							[49]	[44]			
Family	[50, 52, 56]											[54]			
Friends	[52, 55, 56, 59]									[51, 52]		[54, 56]	[56]		

+ Positive association, − negative association, 0 no association; numbers presented in table represent references of included studies for each association

#### Sub-domains of social support

Two studies investigated the relationship between encouragement and PA in adolescent girls longitudinally. One study found a positive association between mother encouragement and adolescent girls’ PA, whilst the other found no association between parent encouragement and adolescent girls PA (see Table 6). One study investigated the relationship between instrumental support and PA, identifying a positive association between parent instrumental support and adolescent girls’ PA. A total of 12 studies investigated the relationship between modelling and adolescent girls’ PA. Of these, five studies identified a positive association, one study found a negative relationship and six studies identified no association between modelling and adolescent girls’ PA. Three studies assessed the relationship between co-participation and PA in adolescent girls. Two studies assessed associations between parent co-participation and adolescent girls’ PA, one of which identified a positive association whilst the other found there to be no association. The last study identified a positive association between friend co-participation and adolescent girls’ PA (see Table 6).

## Discussion

Social support has been identified as a possible modifiable correlate of PA that can be used to inform interventions to enhance PA levels of adolescent girls. This study provided an overview of current evidence of the relationship between different providers and types of social support and adolescent girls’ PA. This adds to previous systematic reviews by presenting the current evidence on all providers and types of support for adolescent girls’ PA, which has not previously been done (see Table 3). We found 21 different providers and 14 different types of social support presented in the literature. Whilst this could mean that there are a substantial number of possible combinations of providers and types of social support, the majority of the studies focused on total social support and modelling from parents, family and friends. There are a number of areas with limited or no research including informational support, watching and talking about PA, and social support from siblings. Whilst this may highlight areas where further research could be needed it also raises questions about whether it is feasible and informative to consider all these possible combinations of support. There may be a need to standardise and refine social support as a construct to improve comparability between types of support and providers within the literature.

### Total social support

With regards the provision of total support, we identified small but significant associations that were similar in magnitude between family and friend total social support with the largest associations for all providers of support and adolescent girls’ PA (*r* = .24). This suggests that both friends and family influence adolescent girls’ PA, however, the small associations suggest that total social support explains only a small amount of the variance in adolescent girls’ PA behaviour.

Our findings both support and contest the findings from a recent meta-analysis by Yao and Rhodes [19] who identified positive associations between parent support and PA in children and adolescents (*r* = .38). We identified more modest effect sizes than Yao and Rhodes [19] for parent support on adolescent girls’ PA (*r* = .19), which aligns more closely with a meta-analysis by Pugliese and Tinsley [27] (*r* = .17). These differences could be attributed to several factors. Yao and Rhodes [19] and Pugliese and Tinsley [27] considered all children and adolescents, whereas we only synthesised associations reported for adolescent girls. It is possible that there are differences in observed effect sizes between older and younger children and boys and girls, although these were not identified as significant moderators in analyses by Yao and Rhodes [19]. Furthermore, the higher observed effect sizes identified by Yao and Rhodes [19] could partially be explained by their analysis procedures, as they corrected effect sizes for sampling and measurement error. This highlights that there is a positive association between parent support and adolescent girls’ PA, although the effect sizes are small.

Our finding that effect sizes were similar in magnitude between parent and friend support variables on adolescent girls’ PA was suprising because the nature of the relationship between children and their parents transforms significantly during adolescence [60]. Adolescents spend less time with their parents and more time with their friends [61], therefore, we anticipated that friends might be better positioned to influence adolescent girls’ PA than parents. Despite the similar effect sizes between parent and friend social support and adolescent girls’ PA, it is likely that ways in which parents and friends provide social support and influence activity levels are different. For example, friends might contribute to positive experiences in physical education or organised physical activities whilst parents could create a foundation for lifelong habits in PA in their children at an early age and provide support for their ongoing participation in PA during adolescence. Further research might investigate these possible mechanisms in more detail. No significant positive associations for teacher support on adolescent girls’ PA were identified. However, only six studies were included in the meta-analysis, which limits our understanding of the relationship between teacher support and PA in adolescent girls. Similar findings were reported in a previous semi-quantitative review [22]. Teachers may, however, play a role in PA behaviour change when given the tools to do so as a recent randomised controlled trial found that teachers mediated the effectiveness of a PA intervention [62].

We also found that PA type moderated effect sizes for total family support with significantly larger associations identified for sports participation compared with MVPA, total PA, after school PA and active travel. This suggests that families may have a greater influence over organised domains of PA. This is perhaps not surprising given that transport, equipment and financial support from parents may be necessary to enable young people to take part in some organised sports. Previous research has also suggested the relationship between social support and PA may vary by type of PA [24]. Other influencing factors (e.g. friends or school infrastructure) may have a stronger role in predicting school-based PA or total PA. Given that girls have been found to take approximately 41–47 % of steps during the school day [63] this is an important consideration for future research.

### Sub-domains of social support

In relation to the different types of social support, meta-analyses showed small but significant associations for encouragement, instrumental support and modelling. For parents, we identified similar effect sizes for encouragement (*r* = .10) and instrumental support (*r* = .17) and we found that co-participation was not significantly related to PA (*r* = .03). This does not support findings from Yao and Rhodes [20] who found that encouragement and co-participation were most strongly related with PA in samples of male and female children and adolescents. This may be explained by differences in sample sizes, number of studies included in the meta-analyses, or it could be that the relationships are weaker when investigating only girls. However, these findings do highlight small but positive associations for encouragement and instrumental support and suggest both are important to a similar extent for adolescent girls’ PA. There were not enough studies to meta-analyse these types of support for friend support variables although Mendonça and colleagues [22] found that friend encouragement and co-participation were most consistently associated with adolescents’ PA. Future research may be needed to quantify the role of different types of friend support on adolescent girls’ PA.

### Longitudinal findings

The results of the longitudinal studies generally reflected cross-sectional findings. Change in total social support from families and friends was consistently related to changes in PA, suggesting that social support is a determinant of PA behaviour in adolescent girls. As previously noted, several study designs were used to assess associations between social support and PA longitudinally. These different study designs may reflect some of the differences in results observed. For example, some analyses used baseline social support to predict follow up PA, whilst others compared changes in social support with changes in PA. We would expect both social support and PA to change over time, therefore, comparing these study designs has limitations. There was less longitudinal evidence for different types of social support (e.g. emotional support) nonetheless results reflected cross-sectional findings in that different types of support seemed to be less consistently associated with PA compared with total support.

### Limitations

The meta-analysis findings should be interpreted with caution for two reasons. Firstly, the analysis did not account for possible indirect effects of social support. The observed effect sizes suggest that social support only explains a small amount of variance in adolescent girls’ PA, however, our analysis did not take account of possible indirect effects of social support on PA. Given that some research has found that self-efficacy [64–66] and competence and value [67] mediates the relationship between social support and PA it is possible that social support indirectly influences PA through self-efficacy and other possible mediating constructs (e.g. enjoyment). Secondly, there was high heterogeneity between studies and some evidence of publication bias. The high heterogeneity statistics may in part be related to sampling error although it is likely that other variables moderated the size of the effects. Our moderator analysis showed that type of PA (e.g. sport, MVPA) predicted the effect size for total family support on adolescent girls’ PA and the relationship between father and daughter PA was moderated by type of PA measures, with subjective measures demonstrating higher effect sizes than objective measures. There were no significant moderators identified for other meta-analyses performed. A previous meta-analysis by Yao and Rhodes [19] carried out moderator analysis and similarly found that subjective measures demonstrated higher effect sizes but they did not test for type of PA.

After performing moderator analysis there was still high heterogeneity between studies so it is likely that other factors not investigated also moderated effect sizes. These high heterogeneity statistics may in part reflect methodological inconsistencies within the literature on associations between social support and PA in adolescent girls. For example, whilst we tried to account for type of PA (e.g. total PA, MVPA, sports participation) and how PA was measured (e.g. subjective or objective measures), alongside other potential moderators, we were not able to account of the vast range of tools used to measure PA (e.g. different subjective measures, accelerometers, see Additional file 4). Whilst these tools all measure PA they are all inherently different with distinct purposes, therefore, it is possible this contributed to variances in the effect sizes and the high heterogeneity statistics. Similarly, social support was measured using various tools. The most commonly reported validated tools used included a scale originally developed for the Amherst Health and Activity Study and later validated [68, 69] and the Activity Support Scale [44]. However, in many cases, these scales were modified for use or authors used non-validated, custom scales to measure social support. This is problematic because this lack of consistency could lead to imprecise measurement, which has been previously recognised as a challenge in the literature [30]. This may also have contributed to variances in the effect sizes and the high levels of heterogeneity identified although our analysis did try to account for this. Furthermore, various analysis techniques were employed across the included studies (e.g. correlations, regressions, growth curve models). Some of these analyses controlled for confounding variables (e.g. ethnicity, age) whilst others did not. This may also have contributed to variances in the effect sizes and high heterogeneity statistics. It was not possible to account for this within our analyses, which is a limitation to our findings.

### Implications

This review has highlighted several implications for future research. Firstly, measurement of social support is inconsistent. With a very high number of possible combinations of types and providers of support identified by this review there is a need to standardise measurement so that more informative comparisons can be made. Secondly, although social support explained only a small amount of variance in adolescent girls’ PA there may be some merit in exploring and testing intervention strategies aimed at increasing different types of social support from friends and families and PA alongside other known determinants of PA in adolescent girls, consistent with a socio-ecological approach to PA behaviour change [15]. In particular, the strongest associations were evident for total social support (or multiple forms of support) from multiple providers of support. This highlights a potential need for interventions to increase girls’ exposure to multiple types and providers of social support. As these findings are specific to adolescent girls, a mirror review should be conducted to understand the relative importance of different types and providers of social support for physical activity in boys.

## Conclusion

Social support from friends, parents and families has a small but positive relationship with PA in adolescent girls and associations were generally similar in magnitude for different providers and types of social support. As the associations were small, other variables may be more important for adolescent girls’ PA. However, the results suggests that overall support from parents and friends, as well as sub-domains of support such as encouragement, instrumental support and modelling, are all associated with adolescent girls’ physical activity. The strongest association was identified for overall social support from all providers of support, which may suggest that being supported by various people in various ways is important for adolescent girls PA. There may, therefore, be promise in including social support components, alongside other known predictors (e.g. strategies to increase self-efficacy), in PA behaviour change interventions targeting adolescent girls. Further research examining the success of such interventions is therefore warranted.

## Abbreviations

MPVA, Moderate-to-vigorous physical activity; PA, Physical activity

## Additional files

Additional file 1: Risk of bias assessment thresholds. (DOCX 14 kb) Additional file 2: Moderators tested in meta-regression. (DOCX 12 kb) Additional file 3: Characteristics of included studies. (DOCX 12 kb) Additional file 4: Data extraction from included studies. (DOCX 233 kb)
