# Supplementary material for: The role of social support on physical activity behaviour in adolescent girls: a systematic review and meta-analysis
Source: Int J Behav Nutr Phys Act. 2016 Jul 7;13:79. doi: 10.1186/s12966-016-0405-7 (PMC4937604; doi:10.1186/s12966-016-0405-7)
Supplement: Additional file 1: — Risk of bias assessment thresholds. (DOCX 14 kb) [file 12966_2016_405_MOESM1_ESM.docx]

**Supplementary file 1** Risk of bias assessment thresholds

| **8-item checklist** | **Criteria** | **Thresholds for criteria** |
| --- | --- | --- |
| Did the study address a clearly focused issue?  Did the authors use an appropriate method to answer their question? | Initial screening questions |  |
| 1. Was the cohort recruited in an acceptable way?   Was the follow-up of the subjects complete enough?  Was the follow-up of the subjects long enough? | Selection bias | Random sample, non-response <30% and loss of follow-up <50% = Low risk  Non-random sample and/or nonresponse ≥ 30% = high risk |
| 1. Was physical activity (outcome) accurately measured to minimize bias? | Physical activity measurement bias | Objective measure (e.g. accelerometer/pedometer) or validated questionnaire (e.g. author references validation study in-text or known validated scale) = low risk  Custom questionnaire or single-item questionnaire = high risk |
| 1. Was social support (exposure) accurately measured to minimize bias? | Social support measurement bias | Self-report previously validated questionnaire = low risk  Custom questionnaire = high risk |
| Have the authors identified all important confounding factors? | Confounding variables | Adjusted for confounders (age, SES, ethnicity) through analysis, stratification, or study design = low risk  Adjusted for some or none of the confounders = High risk |

Assessment checklist collapsed from the CASP Toolkit: <http://media.wix.com/ugd/dded87_36c5c76519f7bf14731ed1985e8e9798.pdf>
