# Supplementary material for: The role of social support on physical activity behaviour in adolescent girls: a systematic review and meta-analysis
Source: Int J Behav Nutr Phys Act. 2016 Jul 7;13:79. doi: 10.1186/s12966-016-0405-7 (PMC4937604; doi:10.1186/s12966-016-0405-7)
Supplement: Additional file 2: — Moderators tested in meta-regression. (DOCX 12 kb) [file 12966_2016_405_MOESM2_ESM.docx]

**Supplementary file 2** Moderators tested in meta-regression

| **Moderator** | **Categories** |
| --- | --- |
| Social support measurement bias | High risk; low risk; or unclear risk |
| Who reported social support | Perceived support (child reported); perceived support (provider reported) |
| Physical activity type | MVPA; Total PA; Sports; Leisure time PA; or Active travel |
| Physical activity measurement type | Objective or subjective |
| Geographical location | USA; Australia/New Zealand; Europe; Asia; South America; or Canada |
| Participant age | 10-12 years; 13-15 years; or 16-19 years |
