# Supplementary material for: The role of social support on physical activity behaviour in adolescent girls: a systematic review and meta-analysis
Source: Int J Behav Nutr Phys Act. 2016 Jul 7;13:79. doi: 10.1186/s12966-016-0405-7 (PMC4937604; doi:10.1186/s12966-016-0405-7)
Supplement: Additional file 3: — Characteristics of included studies. (DOCX 12 kb) [file 12966_2016_405_MOESM3_ESM.docx]

**Supplementary file 3** Characteristics of included studies

| **Characteristic** | **Samples *n* (%)** |
| --- | --- |
| **Geographical Location** | |
| Asia | 7 (8%) |
| Australia | 10 (12%) |
| Canada | 6 (7%) |
| Europe | 13 (15%) |
| South America | 2 (2%) |
| United States | 46 (55%) |
| **Study Design** | |
| Cross-sectional | 68 (81%) |
| Longitudinal | 16 (19%) |
| **Physical Activity Measurement** | |
| Self-report | 60 (71%) |
| Objective | 24 (29%) |
| **Age** |  |
| 10-12 years | 29 (34%) |
| 13-15 years | 44 (52%) |
| 16-19 years | 9 (11%) |
| Not reported (but adolescents) | 2 (2%) |
