# Supplementary material for: The role of social support on physical activity behaviour in adolescent girls: a systematic review and meta-analysis
Source: Int J Behav Nutr Phys Act. 2016 Jul 7;13:79. doi: 10.1186/s12966-016-0405-7 (PMC4937604; doi:10.1186/s12966-016-0405-7)
Supplement: Additional file 4: — Data extraction from included studies. (DOCX 233 kb) [file 12966_2016_405_MOESM4_ESM.docx]

**Supplementary file 4. Data extraction from included studies**

| **Authors, country** | **Sample (number, age)** | **Design** | **Physical activity measure** | **Social support measure** | **Results** | **Effect size *r* included in meta-analysis** |
| --- | --- | --- | --- | --- | --- | --- |
| Anderssen and Wold (1992) Norway ([1](#_ENREF_1)) | N = 406, 13.3 ± 0.3 years | CS | Subjective – Leisure time physical activity (used in WHO cross-national surveys) | Does not reference scale source – looks like custom scale [child report] | Mother modelling *r* = .14 | .14 |
|  |  |  |  |  | Father modelling *r* = .14 | .14 |
|  |  |  |  |  | Best friend modelling *r* = .31 | .31 |
|  |  |  |  |  | Mother encourage *r* = .28 | .28 |
|  |  |  |  |  | Father encourage *r* = .25 | .25 |
|  |  |  |  |  | Parent encourage *r* = .22 | .22 |
|  |  |  |  |  | Friend encourage *r* = .30  (not enough studies for meta-analysis) | N/A |
|  |  |  |  |  | Parent instrumental support β = .19 | .19 |
|  |  |  |  |  | Total encouragement β = .19 | .19 |
|  |  |  |  |  | Total modelling β = .17 (only study – not enough for meta-analysis) | .17 |
| Baskin (2013)  USA ([2](#_ENREF_2)) | N = 62, 13.8 ± 1.4 | CS | Objective – Accelerometers and activity log | Sallis scale [parent report] | Parent social support on child MVPA: Estimate 1.645, p <0.0001 | .474 |
| Bauer (2008)  USA ([3](#_ENREF_3)) | N = 395, 12.8 ± 0.8 and 15.8 ± 0.6 years | Longitudinal | Subjective – modified version of the LTEQ | Does not reference scale source – looks like custom scale [child report] | Encouragement among younger females to be physically active from their mothers was associated with greater hours of MVPA five years later (p < .01 for trend). Adolescents perceptions of mother modelling was not related to MVPA after 5 years but father modelling was related to MVPA among older females. | N/A |
| Bauer (2011)  USA ([4](#_ENREF_4)) | N = 253, 15.7 years | CS | Subjective – 3DPAR (MVPA) | Modelling - 3 item scale Godin and Shephard (1985) [parent report] and Sallis scale for total support [parent report] | Estimate/p-value |  |
|  |  |  |  |  | Parent modelling 0.40, *p*= .047 | .126 |
|  |  |  |  |  | Family support 0.28, *p* = .169 | .087 |
| Beets (2007) A USA ([5](#_ENREF_5)) | N = 259, 15.5 ± 1.2 years | CS | Subjective – HBSC tool and 2 others | Sallis scale [child report] | Peer support β = .42 | .42 |
| Beets (2007) B USA ([6](#_ENREF_6)) | N = 39, 10 ± 0.8 years | CS | Pedometer (7 days) | Modified Sallis and Activity Support scale [parent report] | Mother encourage β = .102 | .102 |
|  |  |  |  |  | Mother watch β = .100 (only 1 – not enough for meta-analysis) | .100 |
|  |  |  |  |  | Mother co-participation β = -.073 | -.073 |
| Bradley (2011) USA ([7](#_ENREF_7)) | N = 405, 9-15 years at baseline | CS and Longitudinal | Accelerometer (7 days) | Does not reference scale source. Modelling and social support [parent report]. | Parent encouragement ES = 0.02 | .02 |
|  |  |  |  |  | Parent modelling = 0.09 | .09 |
|  |  |  |  |  | Parent co-participation = 0.05 | .05 |
|  |  |  |  |  | Parent transport ES = 0.14 | .14 |
| Brown (1989) Canada ([8](#_ENREF_8)) | N = 376, 13-19 years | CS | Subjective – Assessed participation in intramural activity, interschool sport and community sport (number of years participate in).  Unclear of specific differences so took an average of the three for effect size. | Encouragement, support and modelling [child report]. | *r* = Intramural, interschool, community |  |
|  |  |  |  |  | Total encouragement *r* = .32, .34, .17 (mean *r* = .276) | .276 |
|  |  |  |  |  | Total support *r* = .28, .28, .13 (mean *r* = .23) | .23 |
|  |  |  |  |  | Father encouragement *r* = .18, .24, .18 (mean *r* = .20) | .20 |
|  |  |  |  |  | Mother encouragement *r* = .14, .19, .10 (mean *r* = .143) | .143 |
|  |  |  |  |  | Father support β = .197, β = .209, *r* = .19 (mean β = .203) | .203 |
|  |  |  |  |  | Mother support *r* = .20, .19, .15 (mean *r* = .18) | .18 |
|  |  |  |  |  | Father modelling *r* = .10, .12, .15 (mean *r* = .123) | .123 |
|  |  |  |  |  | Mother modelling *r* = .08, β = .116, β = .202 (mean β =.159) | .159 |
|  |  |  |  |  | Male friend encouragement β = .147, *r* = .20, *r =* .03 | Not enough studies for meta-analysis |
|  |  |  |  |  | Female friend encouragement *r* = .14, β = .158, *r* = .12 |  |
|  |  |  |  |  | Male friend support *r* = .20, .21, .08 |  |
|  |  |  |  |  | Female friend support *r* = .19, .22, .11 |  |
| Bungum (1997) USA ([9](#_ENREF_9)) | N = 852, 14-18 years | CS | Subjective – Standford Physical Activity Recall Questionnaire | Peer modelling, parent modelling, friend support, family support (derived from Sallis, 1987) [child report]. | No significant associations for all participants (some significant when separated by ethnic group –non-significant associations not reported) | N/A |
| Burns (2014)  UK ([10](#_ENREF_10)) | N = 871, 15.28 ± 1.8 years | CS | Stages of change model (Marcus et al., 2003) | Sallis scale for friend social support [child report]. | Friend support β = .17 | .17 |
| Canfield (2012) USA ([11](#_ENREF_11)) | N = 265, 13.2 years | CS | Subjective – modified from PAQ-A and Sallis Amherst study | Parental encouragement and modelling, unclear where derived from [child report]. | Parent modelling β = .201 | .201 |
|  |  |  |  |  | Parent encouragement β = -.126 | -.126 |
| Cheng (2014)  Brazil ([12](#_ENREF_12)) | N = 1336, 16.4 ± 1.2 years | CS | Subjective (MVPA) – previously used, cites  validation study | Previously used support scale – cites validation study. Unclear on modelling [child report]. | Friend modelling: β = .07 | .07 |
|  |  |  |  |  | Father modelling: β = -.01 | -.01 |
|  |  |  |  |  | Mother modelling: β = .08 | .08 |
|  |  |  |  |  | Friend social support: β = .20 | .20 |
|  |  |  |  |  | Parent social support: β = .17 | .17 |
| Crawford (2010) Australia ([13](#_ENREF_13)) | N = 173, 10-12 years | Longitudinal | Accelerometer (8 days) | Parents for social support, siblings for co-participation (adapted from Sallis) [parent report]. | Parent co-participation b = 1.73, *p* < .05, (0.45-3.02) and father role modelling b – 0.39, *p* < .05, (0.01-0.76) | |
| Crimi (2009)  USA ([14](#_ENREF_14)) | N = 90, 13.14 ± 2.64 years | CS | PAQ-C and PAQ-A | CPAC [child report]. | Parent role modelling *r* = .23 | .23 |
|  |  |  |  |  | Parent support *r* = .32 | .32 |
|  |  |  |  |  | Parent encouragement *r* = .31 | .31 |
| Davison (2004) USA ([15](#_ENREF_15)) | N= 92, 12.5 ± 0.8 years | CS | CPA scale, Activity checklist and the PA subscale of the Physical Self-Description questionnaire | ACT scale [child report]. | The percentage of girls who were highly active was significantly higher when one parent provided a high level of support in contrast to no parents providing a high level of support (OR = 0.14, CI = 0.03-0.60). | N/A |
| Davison (2006) USA ([16](#_ENREF_16)) | N = 174, 9-11 years at baseline | Longitudinal | Activity checklist and CPA scale | ACT scale [parent report]. | Parent support at 11 significantly predicted PA at age 11. Parental support at age 11 mediated the association between perceived competence at 9 years and PA at 11 years. No association was identified between parental support for girls at age 9 years and perceived competence at 11 years but parental support for girls at 9 years predicted support at 11 years which in turn predicted PA at 11 years. | N/A |
| De Farias (2014)  Brazil ([17](#_ENREF_17)) | N = 1653, 16.4 ± 1.19 | CS | Subjective – cites validation study  MVPA – meets vs does not meet guidelines | Unclear scale [child report]. | Parent social support OR: 2.69 (2.13, 3.39) 95% CI | .104 |
|  |  |  |  |  | Friend social support OR: 2.65 (1.94, 3.61) 95% CI | .259 |
| Deflandre (2001) France ([18](#_ENREF_18)) | N = 22, 17 ± 0.9 years | CS | Subjective – Weekly MVPA (also notes ‘physical and sports activity’ but used MVPA in analysis). | Does not reference scale source – looks like custom scale. Measures modelling, encouragement and total support [child report]. | Father modelling *r* = .35 | .35 |
|  |  |  |  |  | Mother modelling *r* = .21 | .21 |
|  |  |  |  |  | Sibling modelling *r* = .27 | .27 |
|  |  |  |  |  | Friend modelling *r* = -.22 | .22 |
|  |  |  |  |  | Total encouragement *r* = .16 | .16 |
|  |  |  |  |  | Parent support *r* = .31 | .31 |
| Dewar (2013)  Australia ([19](#_ENREF_19)) | N = 235, 13.2 ± 0.4 years | CS and longitudinal | Objective – accelerometer (MVPA) | Unclear – looks like custom scale [child report]. | Parent support baseline PA *r* = -.01 | -.01 |
|  |  |  |  |  | Parent support 12 month PA *r* = .08 |  |
| DiLorenzo (1998) USA ([20](#_ENREF_20)) | N = 54, 11.2 ± 0.7 years | Longitudinal and CS | PAI | CPAC – parent modelling, total support [child report]  Parent modelling [parent report]. | CS Mother modelling F(4,49) = 5.36, *p* = .02 (inversely) | -.316 |
|  |  |  |  |  | CS Total support F(4,49) = 2.33, *p* = .13 | .209 |
|  |  |  |  |  | Longitudinal results (extent to which phase 1 support variables predict PA at phase 2 – 3 years later: The only support variable that entered into the model was mother modelling which was inversely related F(5,36) = 2.81, *p* = .10 |  |
| Dishman (2010) USA ([21](#_ENREF_21)) | N = 971, 10-16 years | CS and Longitudinal | Accelerometers (7 days)  Daily METs weighted minutes of MVPA | Sallis scale [child report]. | CS association between social support and PA in 6^th^ grade *r* = .12 | .12 |
|  |  |  |  |  | There was not a significant direct path between social support and PA (β = -.05, Se = .08, *p* =.548). The authors attributed this to the covariance of self-efficacy and perceived social support (β = .54, SE = .04, *p* <.001)  *(Between 6^th^ and 8^th^ grade)* |  |
| Dishman (2009) USA ([22](#_ENREF_22)) | N = 195, 13.6 ± 0.6 years | CS and Longitudinal | 3DPAR | Modified Social Provisions Scale (Cutrona & Russell, 1987) [child report]. | CS association between social support and PA β = .52 | .52 |
|  |  |  |  |  | Change in social support on change in physical activity β = .21 | .21 |
| Dollman (2009) Australia ([23](#_ENREF_23)) | N = 827, 11.97 ± 1.41 | CS | PAQ-A | CPAC and modelling [parent report] (Prochaska, 1997) | Parent support β = .32 | .32 |
| Dowda (2007) USA ([24](#_ENREF_24)) | N = 421, 13.6 ± 0.7 years | CS and Longitudinal | 3DPAR | Sallis scale (2002) [child report]. | Family support 8^th^ grade *r* = .26 | .26 |
|  |  |  |  |  | Family support 9^th^ grade *r* = .23 | .23 |
|  |  |  |  |  | Family support 12^th^ grade *r* = .26 | .26 |
|  |  |  |  |  | Longitudinal findings: Models indicate that girls who had higher family support at 8^th^ grade had higher total MET scores and change in PA is significantly correlates with change in family support. |  |
| Duncan (2007) USA ([25](#_ENREF_25)) | N = 186, 12.05 ± 1.63 years | CS and Longitudinal | Pedometers (7 days) and two single item questions based on Youth Risk Behavior Survey | Parent modelling (single item question) [parent report]  Parent and friend support (Sallis 2002) and friend modelling [child report] | CS Friend modelling | .26 |
|  |  |  |  |  | Girls with more physically active friends had less of a decline in PA from ages 12 to 17. There was also an effect of time 1 friend PA on the slope, such that girls with initially more active friends had a greater decline in PA from ages 12 to 17. The authors attributed this to a change score effect. E.g. simultaneous estimation of change in the variable would indicate a decline in this covariate over time. |  |
| Edwardson (2013)  UK ([26](#_ENREF_26)) | N = 142, 12-16 years | CS | Objective – accelerometer | Activity Support Scale [child report]. | Total sibling support *r* = .29 (not enough studies to perform meta-analysis) | N/A |
| Eime (2013)  Australia ([27](#_ENREF_27)) | N = 732, 13.6 ± 1.96 years | CS | Subjective – single item question on sports involvement (yes/no response) | Sallis scale [child report]. | Family support β = .39, .54, .57. Mean β = .50 | .50 |
|  |  |  |  |  | Friend support β = .07, .04, .09. Mean β = .067 | .067 |
| Frenn (2005)  USA ([28](#_ENREF_28)) | N = 52, 12.75 years | CS | CAAL | Family, friend and classmate support (Garcia, 1995) [child report]. | Total support β = .566 | .566 |
| Graham (2014)  USA ([29](#_ENREF_29)) | N = 356, 15.8 ± 1.2 years | CS and Longitudinal | Subjective – 3DPAR (average daily 30 minute blocks spent in MVPA) | Previously validated scale – references New Moves study [child report]. | CS Family support β = .55 | .55 |
|  |  |  |  |  | CS Friend support β = .41 | .41 |
|  |  |  |  |  | CS Teacher support β = .48 | .48 |
|  |  |  |  |  | CS Parent modelling β = .51 | .51 |
|  |  |  |  |  | CS Friend modelling β = .56 | .56 |
|  |  |  |  |  | Change in family support on MVPA at follow up β = .30 | .30 |
|  |  |  |  |  | Change in friend support on MVPA at follow up β = .31 | .31 |
|  |  |  |  |  | Change in teacher support on MVPA at follow up β = .01 | .01 |
|  |  |  |  |  | Change in parent modelling on MVPA at follow up β = .29 | .29 |
|  |  |  |  |  | Change in friend modelling on MVPA at follow up β = .21 | .21 |
| Graham (2014) B  USA ([30](#_ENREF_30)) | N = 1486, 14.4 ± 2 years | CS | Subjective – modified LTEQ (MVPA) | Seems to be a combination of previously used and custom scales  Parent modelling, co-participation and instrumental support [parent report]  Family and friend support [child report]  Friend modelling [Friend report] | Parent modelling β = .021 | .021 |
|  |  |  |  |  | Mother modelling β = -.125 | -.125 |
|  |  |  |  |  | Father modelling β = .216 | .216 |
|  |  |  |  |  | Parent co-participation on β = -.118 | -.118 |
|  |  |  |  |  | Parent instrumental support β = .205 | .205 |
|  |  |  |  |  | Family support *p* = .727 | .009 |
|  |  |  |  |  | Friend support *p* = .025 | .058 |
|  |  |  |  |  | Male friend modelling β = .330 (not enough studies for meta-analysis) | .330 |
|  |  |  |  |  | Female friend modelling β = -.317 (not enough studies for meta-analysis) | -.317 |
| Gregson (1986) UK ([31](#_ENREF_31)) | N = 130, 15-16 years | CS | Self-reported number of hours per week playing sport | Asks about mother and father involvement in sport [child report]. | Mother modelling β = .084 | .084 |
|  |  |  |  |  | Father modelling β = .139 | .139 |
| He (2013)  Japan ([32](#_ENREF_32)) | N = 280, 13.44 ± 0.93 years | CS | Subjective – unclear on scale or if validated (lunch-time and after school PA) | Assesses family and friend support, no validation information [child report]. | Family support *(on after school PA)* β = .13 | .13 |
|  |  |  |  |  | Friend support *(on after school PA)* β = .16 | .16 |
| Huang (2013)  China ([33](#_ENREF_33)) | N = 146, 11.2 ± 0.9 years | CS | Subjective – CLASS-C (MVPA) | Assesses family and friend support [child report] and parent role modelling [parent report], no validation information. | B (95%CI)  Friend support = 0.95 (0.35, 1.54), *p* < 0.01  Family support and parent modelling not significant – no data able to be extracted. | .213 |
| Jackson (2013)  UK ([34](#_ENREF_34)) | N = 244, 12.8 ± 0.9 years | CS | Subjective – PAQ-A (total PA) | Sallis scale for parent total support [child report]. | Parent support β = .51 | .51 |
| Jago (2011)  UK ([35](#_ENREF_35)) | N = 330, 10-11 years | CS | Accelerometers (≥ 3 days) | ACT scale  Guiding support defined as ‘rules for PA’ [child report]. | Mother logistic support on MVPA coefficient 1.7 (-0.50 to 3.93) 95% CI, *p* = .13 | .084 |
|  |  |  |  |  | Guiding support on MVPA coefficient 1.2 (0.04 to 2.36) 95% CI, *p* = 0.04  (not enough studies for meta-analysis) |  |
| Jago (2014)  UK ([36](#_ENREF_36)) | N = 215, 10 years | CS | Objective – accelerometer for mean minutes of weekday MVPA and after school MVPA (5 days) | Revised parent ACTs [child report]. | Coefficient (95% CI) *p* |  |
|  |  |  |  |  | Mother modelling 0.28 (-4.45, 5.01), *p* = .903 | .008 |
|  |  |  |  |  | Mother logistic support 2.7 (-1.44, 6.83), *p* = .188 | .090 |
|  |  |  |  |  | Father modelling -1.05 (-4.96, 2.85), *p* = .579 | -.038 |
|  |  |  |  |  | Father logistic support 0.41 (-3.79, 4.60), *p* = .841 | .014 |
| Kahn (2008)  USA ([37](#_ENREF_37)) | N = 7237, 10-16 years at baseline | CS and Longitudinal | Subjective – assessed time spent during past year in 18 PA’s to estimate total PA | Mother modelling [mother report]. Unclear where scale derived from. | Mother modelling β = 0.130 for cross-sectional associations at baseline (*n* = 7237) | .130 |
|  |  |  |  |  | Evaluated whether baseline support modelling predicted follow-up PA. None of the variables significantly predicted follow-up PA – results not reported. |  |
| Kelly (2010)  USA ([38](#_ENREF_38)) | N = 1180, 6^th^ grade | CS | Accelerometer (6 days) | Sallis scale for family and friend support [child report]. | Family support Hispanic MVPA (n = 185) b = 0.008, *p* = .418 | .06 |
|  |  |  |  |  | Friend support Hispanic MVPA (n = 185) b = 0.027, *p* = .061 | .138 |
|  |  |  |  |  | Family support black MVPA (n = 289), *r* = .07 | .07 |
|  |  |  |  |  | Friend support black MVPA (n = 289), b = 0.026, *p* = .006 | .161 |
|  |  |  |  |  | Family support white MVPA (n = 706), b = -0.001, *p* = .879 | .006 |
|  |  |  |  |  | Friend support white MVPA (n = 706), b = 0.020, *p* =.010 | .097 |
| Keresztes (2008) Hungary ([39](#_ENREF_39)) | N = 247, 12.2 ± 1.2 years | CS | Subjective – Leisure Time PA | Modelling scale based on other studies [child report]. | Parent modelling OR 2.72 (1.15-6.44) 95% CI | .266 |
|  |  |  |  |  | Sibling modelling OR 2.22 (1.24-3.97), 95% CI | .215 |
|  |  |  |  |  | Classmates modelling OR 2.83 (1.58-5.05), 95% CI  (not enough studies for meta-analysis) | N/A |
|  |  |  |  |  | Friend modelling OR 2.48 (1.44-4.36), 95% CI | .243 |
|  |  |  |  |  | Boy/girlfriends PA OR 1.78 (1.15-3.30), 95% CI  (not enough studies for meta-analysis) | N/A |
| Kirby (2011)  UK ([40](#_ENREF_40)) | N = 328, P7 at baseline | Longitudinal | PAQ-C | Sallis scale [child report]. | Friend support P7 OR 1.31 (0.68, 2.53), 95% CI | .074 |
|  |  |  |  |  | Friend support S2 OR 1.51 (0.78, 2.90), 95% CI | .113 |
|  |  |  |  |  | Friend support S4 OR 2.86 (1.55, 5.27), 95% CI | .278 |
|  |  |  |  |  | Father support P7 OR 1.58 (0.82, 3.07), 95% CI | .125 |
|  |  |  |  |  | Father support S2 OR 1.11 (0.53, 2.34), 95% CI | .029 |
|  |  |  |  |  | Father support S4 OR 1.48 (0.72, 3.03), 95% CI | .107 |
|  |  |  |  |  | Mother support P7 OR 1.80 (0.83, 3.93), 95% CI | .16 |
|  |  |  |  |  | Mother support S2 OR 1.70 (1.11, 2.61) | .145 |
|  |  |  |  |  | Mother support S4 OR 1.39 (0.54, 3.58) | .09 |
| Kitzman-Ulrich (2010)  USA ([41](#_ENREF_41)) | N = 375, 11.4 ± 0.7 years | CS | Accelerometers (7 days) | Social Support for Exercise Scales (Sallis, 1987) [child report]. | Family support *r* = -.03 | -.03 |
|  |  |  |  |  | Friend support *r* = .07 | .07 |
| Krishnamoorthy (2002)  USA ([42](#_ENREF_42)) | N = 101, 13 ± 1 years | CS | Accelerometers (7 days) and 7DPAR  Used accelerometer data | Social Support for Exercise Scales (Sallis, 1987) [child report] and 7DPAR for modelling [primary caregiver report]. | Primary caregiver modelling *r* = .13  (not enough studies for meta-analysis) | .13 |
|  |  |  |  |  | Family support *r* = .05 (7DPAR)/ -.20 (accelerometer) | -.20 |
|  |  |  |  |  | Friend support *r* = .03 (7DPAR)/ -.03 (accelerometer) | -.03 |
| Kuo (2007)  USA ([43](#_ENREF_43)) | N = 221, 13-15 years | CS | 7DPAR | Sallis scale and family involvement in PA’s scale [child report]. | Family support significantly predicted PA at multivariate *p* = .03 (associated with A in bivariate *r* = .18) | .146 |
|  |  |  |  |  | Family modelling at multivariate *p* = .04, (*r* = .17 for bivariate) | .138 |
| Kurc (2009)  Canada ([44](#_ENREF_44)) | N = 11,017, 14-19 years | CS | Self-report minutes of moderate and VPA in last 7 days: kilocalories per kg of body weight/day. Categorised into low active and active. | Parent support. No information on where scale derived from – seems to be custom scale. Categorised into low support or socially supported [child report]. | Low parent social support on low active OR 0.71 (0.58, 0.89) | .09 |
| Lee (2010)  Singapore ([45](#_ENREF_45)) | N = 895, 14.4 ± 1.1 years | CS | 3DPAR | Sallis scale [child report]. | Parent support β = .129 | .129 |
| Leggett (2012)  Canada ([46](#_ENREF_46)) | N = 15736, grade 9-12 | CS | Subjective - Based on SHAPES to assess how much PA they did in the previous week | Parent encouragement and how many of their closest friends are physically active. No information on where scales derived from – seem to be custom [child report]. | Parent encouragement active vs inactive OR 1.66 (1.50, 1.83), 95% CI | .138 |
|  |  |  |  |  | 3-5 active friends active vs inactive OR 2.14 (1.94, 2.36) 95% CI (modelling) | .205 |
| Lenhart (2014)  USA ([47](#_ENREF_47)) | N = 168, 4^th^/6^th^/8^th^ grade | CS | Subjective – PAQ-C (Total PA) | The Social Support for Physical Activity Measure [child report]. | Total support OR 9.03 (1.95, 41.75) 95% CI | .519 |
|  |  |  |  |  | Parent modelling OR 1.95 (0.98, 3.85) 95% CI | .181 |
| Leslie (2010)  Australia ([48](#_ENREF_48)) | N = 1504, 11.4 ± 0.8 years | CS | Subjective – reported on how they usually travelled to and from school (active travel)  As very similar measures used most modest results for each provider | Sallis scale (2002) for family and friend support [child report]. | Odds of active travel to school with high family support OR 0.81 (0.58-1.13), 95% CI | -.058 |
|  |  |  |  |  | Odds of active travel from school with high family support OR 0.70 (0.50-0.99) 95% CI | -.098 |
|  |  |  |  |  | Odds of active travel to school with high friend support OR 1.27 (0.94-1.73) 95% CI | .066 |
|  |  |  |  |  | Odds of active travel from school with high friend support OR 1.36 (0.99-1.86) 95% CI | .085 |
| Ling (2014)  USA ([49](#_ENREF_49)) | N = 509, 11.76 ± 0.76 | CS | Objective – Accelerometer (7 days) | Total support – study aimed to validated scale [child report]. | Total support on MVPA *r* = .13 | .13 |
| Lubans (2009)  Australia ([50](#_ENREF_50)) | N = 72, 14.2 ± 0.7 years | CS | Pedometer (4 school days) – mean steps/day | Sallis scale (friends) [child report]. | Friend support *r* = .260 | .260 |
| McGuire (2002)  USA ([51](#_ENREF_51)) | N = 477, age not reported (adolescents) | CS | LTEQ (Total PA) | Parent encouragement [child report]  Parent modelling and encouragement [parent report]. No information on where scales derived from – look like custom scales | Parent modelling partial *r* = .06, *p* = .456 | .034 |
|  |  |  |  |  | Parent reported encouragement partial *r* = .15, *p* < .001 *r* = .152) and adolescent reported parent encouragement partial *r* = .15, *p* < .001 (*r* = .137). Mean of constructs *r* = .1445 | .1445 |
| Morgan (2003)  USA ([52](#_ENREF_52)) | N = 99, 11.6 ± 0.6 years | CS | 7DPAR | Teacher modelling, parent modelling, parent support and parent transport. Adapted from previously used scales [child report]. | Parent transport partial *r* = .217, *p* < .05 | .198 |
| Morrissey (2012)  USA ([53](#_ENREF_53)) | N = 144, 13 ± 0.26 years | CS | Accelerometer (up to 5 days) | Sallis scale [child report]. | B, SE, *p*, partial *r* squared |  |
|  |  |  |  |  | Friend support on non-school MVPA *r* = .12 | .12 |
|  |  |  |  |  | Family support on non-school MVPA B = 7.93, SE 2.41, *p* = .001, partial *r* squared = .081 | .271 |
|  |  |  |  |  | Family praise on non-school MVPA B = 5.80, SE 1.85, *p* = .002, partial *r* squared .070  (not enough studies for meta-analysis) | N/A |
| Motl (2007)  USA ([54](#_ENREF_54)) | N = 1655, 17.7 ± 0.6 years | CS | 3DPAR | Social provisions scale [child report]. | Social support β = .28 | .28 |
| O’Loughlin (1999)  Canada ([55](#_ENREF_55)) | N = 1141, 9-13 years | CS | 7-day recall adapted from the self-reported weekly activity checklist (Sallis) to categorise into inactive, moderately active and frequently active. Sport team participation and participation in organized sports outside school. | Parent role modelling and support for PA – previously used scales by author although unclear if validated [unclear if support parent or child report – both completed surveys]. | Sports outside school  Mother encourages sports OR 1.6 (1.2-2.6), 95% CI | .128 |
|  |  |  |  |  | Mother does sports OR 1.6 (1.1-2.1) | .128 |
| Patnode (2010)  USA ([56](#_ENREF_56)) | N = 145, 15.3 ± 1.7 years | CS | Accelerometer (7 days) (MVPA) | Social support [child report]. No information on where scale derived from but looks like the Sallis scale. Modelling – IPAQ [Parent report]. | Parent support *r* = .09 | .09 |
|  |  |  |  |  | Friend support *r* = .13 | .13 |
|  |  |  |  |  | Parent modelling *r* = .03 | .03 |
| Pearson (2009)  Australia ([57](#_ENREF_57)) | N = 421, 11.2 ± 0.6 years | CS | Accelerometer (8 days) | No information on where scale derived from. Measures co-participation, transport and financial support [parent report]. | Parent co-participation  OR 1.36 (0.90-2.07) 95% CI | .084 |
|  |  |  |  |  | Parent transport  OR 1.42 (0.98-2.07) 95% CI | .096 |
|  |  |  |  |  | Parent financial  OR 1.56 (1.08-2.26) 95% CI | .122 |
| Pis (2006)  USA ([58](#_ENREF_58)) | N = 48, grades 5 and 6 | CS | GLTEQ | Social Influences Scale [child report]. | Total support β = -.37 | -.37 |
| Price (2008)  USA ([59](#_ENREF_59)) | N = 1000, aged 9-12 years | CS | Subjective – Weight bearing PA. Looks like custom scale. | Friend modelling and PA co-participation [child report]. Encouragement and modelling [parent report]. No information on where scales derived from – looks like custom scales. | Parent modelling β = .03 | .03 |
|  |  |  |  |  | Parent talking β = .06  (not enough studies for meta-analysis) | .06 |
|  |  |  |  |  | Parent co-participation β = .13 | .13 |
|  |  |  |  |  | Friend modelling β = .09 | .09 |
| Ramanathan (2013)  India ([60](#_ENREF_60)) | N = 69, 15.3 ± 1.1 years | CS | Subjective – PAQ-A (total PA) | Perceived parental attitudes scale (Welk, 2003) [child report]. | Total parent support β = .01 (-.23, .28) | .01 |
| Raudsepp (2000)  Estonia ([61](#_ENREF_61)) | N = 191, 13.8 ± 0.5 years | CS | Subjective – 7 day PA recall (Sallis et al., 1985) | Parent and sibling modelling – 7 day PA recall [parent and sibling report] (Sallis et al., 1985) | Brother modelling β = .09 (not enough  (not enough studies for meta-analysis) | N/A |
|  |  |  |  |  | Mother modelling β = .12 | .12 |
|  |  |  |  |  | Sister modelling β = .17  (not enough studies for meta-analysis) | N/A |
|  |  |  |  |  | Father modelling β = .20 | .20 |
| Raudsepp (2006)  Estonia ([62](#_ENREF_62)) | N = 158, 13.8 years | CS | Subjective – 7 day PA recall (Sallis et al., 1985) | ACT [Parent report]. | Father logistic *r* = .32 | .32 |
|  |  |  |  |  | Father modelling *r* = .35 | .35 |
|  |  |  |  |  | Mother logistic *r* = .31 | .31 |
|  |  |  |  |  | Mother modelling *r* = .33 | .33 |
| Raudsepp (2008)  Estonia ([63](#_ENREF_63)) | N = 193, 12.6 ± 0.5 years | CS and longitudinal | 3DPAR | Friend social support [child report] (Duncan et al., 2005)  Friend modelling – 3DPAR [friend report]. | Friend support baseline with PA baseline β = .25 | .25 |
|  |  |  |  |  | Longitudinal findings: Change in PA was significantly and directly related to change in friend support β = .51 |  |
|  |  |  |  |  | 4 month modelling with 16 month PA -.34, 1.45, *p* = not significant |  |
| Reynolds (1990) USA ([64](#_ENREF_64)) | N = 355, 14 to 16 years | Longitudinal and CS | Self-reported total PA activity checklist (previously validated) | Modelling measured at 4 months post baseline. No information on where scales derived from – looks like custom scale [child report]. | B, F, P –value  (note that negative coefficient represents positive relationship in this analyses) |  |
|  |  |  |  |  | CS 4 month PA with modelling (all providers) -1.00, 12.04, *p* = .0007  (not enough studies for meta-analysis) | N/A |
|  |  |  |  |  | 4 month modelling with 16 month PA -0.34, 1.45, *p* = not significant |  |
| Sallis (1999)  USA ([65](#_ENREF_65)) | N = 229 grades 4-6, N = 208 grades 7-9, N = 210 grades 10-12 | CS | 11-item scale to assess total PA | Social support scales developed [unclear if parent or child report]. | Family support grades 4-6 partial *r* = .292 (*p* < .001) | .216 |
|  |  |  |  |  | Family support grades 7-9 partial *r* = .161 (*p* < .05) | .136 |
|  |  |  |  |  | Family support grades 10-12 partial *r* = .375 (*p* < .001) | .225 |
| Sallis (2002)  USA ([66](#_ENREF_66)) | N = 99 in grades 4-6, N = 126 in grades 7-9 and N = 105 in grades 10-12 | CS | Accelerometer (7 days) | Sallis scale for family and friend support, parent modelling [parent report] | Friend support grade 4-6 β = .16 | .16 |
|  |  |  |  |  | Family support grade 4-6 β = .08 | .08 |
|  |  |  |  |  | Friend support grade 7-9 β = .23 | .23 |
|  |  |  |  |  | Family support grade 7-9 β = .02 | .02 |
|  |  |  |  |  | Friend support grade 10-12 β = .01 | .01 |
|  |  |  |  |  | Family support grade 10-12 β = .20 | .20 |
|  |  |  |  |  | Adult modelling grade 4-6 *r* = .09  (not enough studies for meta-analysis) | .09 |
|  |  |  |  |  | Adult modelling grade 7-9 *r* = -.10  (not enough studies for meta-analysis) | -.10 |
|  |  |  |  |  | Adult modelling grade 10-12 *r* = .004  (not enough studies for meta-analysis) | .004 |
| Saunders (2004)  USA ([67](#_ENREF_67)) | N = 1797, 13.6 ± 0.6 years | CS | Subjective. MVPA measured using 3DPAR. Team sport involvement measured by 2 item scale. | Sallis scale [child report]. | Family support for MPVA Y = 0.10 | .10 |
| Sawka (2014)  Canada ([68](#_ENREF_68)) | N = 535, 11 to 15 years | CS | Subjective – HBSC tool | Modelling – Linked nominated friends PA levels [friend report].  *Paper also measures and reports social support association but measure is not social support for PA so results not included* | Proportion of active close friends OR 1.14 (1.02-1.27) | .036 |
| Schofield (2007)  Australia ([69](#_ENREF_69)) | N = 318, 16 ± 0.8 years | CS | Pedometers (4 days) | Step counts of three nominated friends (pedometer 4 days) | First nominated friend β = .41 | .41 |
|  |  |  |  |  | Second nominated friend β = .16 | .16 |
|  |  |  |  |  | Third nominated friend β = .12 | .12 |
| Shokrvash (2013)  Iran ([70](#_ENREF_70)) | N = 207, 12.93 ± 0.49 | CS | Subjective – modified version of APARQ | Seems to be custom family support scale [child report]. | Informational family support OR 1.10 (0.80-1.23)  (not enough studies for meta-analysis) | N/A |
|  |  |  |  |  | Emotional family support OR 1.02 (0.67-0.99)  (not enough studies for meta-analysis) | N/A |
|  |  |  |  |  | Instrumental family support OR 1.11 (1.02-1.24)  (not enough studies for meta-analysis) | N/A |
| Shafer (2012)  USA ([71](#_ENREF_71)) | N = 55, 18.7 ± 0.7 years | CS | 7DPAR | Social Support for Exercise Scale (Sallis, 1987) [child report] | Friend support for moderate PA *r* = -.056; Friend support for hard PA *r* = -.237; Friend support for very hard PA *r* = .130; Friend support for total PA β = -.037 (used β value) | -.037 |
|  |  |  |  |  | Family support for moderate PA *r* = .090; Family support for hard PA *r* = .133; Family support for very hard PA *r* = .136; Family support for total PA *r* = .094. Mean *r* = .120 | .120 |
| Sharma (2009)  USA ([72](#_ENREF_72)) | N = 718, 11.6 ± 0.4 years | CS | Subjective – SAPAC for total physical activity and COPA for weight bearing PA | COPA for family and friend social support: co-participation and encouragement to be active [child report]. | Family encouragement mean mins/day PA *r* = .19  (not enough studies for meta-analysis) | N/A |
|  |  |  |  |  | Family co-participation mean mins/day PA *r* = .19  (not enough studies for meta-analysis) | N/A |
|  |  |  |  |  | Friend encouragement mean mins/day PA *r* = .21 | N/A |
|  |  |  |  |  | Friend co-participation mean mins/day PA *r* = .24 | N/A |
|  |  |  |  |  | Total support on LTPA *r* = .26 | .26 |
| Taymoori (2010)  Iran ([73](#_ENREF_73)) | N = 558, 14.43 ± 1.6 years | CS | CAAL (minor modifications) for total PA (mins/week) | Social support scale (Garcia et al., 1995) [child report]. | Mother support *r* = .26 | .26 |
|  |  |  |  |  | Father support *r* = .14 | .14 |
|  |  |  |  |  | Sibling support *r* = .10  (not enough studies for meta-analysis) | N/A |
|  |  |  |  |  | Friend support *r* = .09 | .09 |
| Thompson (2013)  USA ([74](#_ENREF_74)) | N = 39, 14.2 ± 1.6 years | CS | Modified version of the APARQ | FSS and SIS [child report]. | Total family support *r* = .167 | .167 |
|  |  |  |  |  | Total social support *r* = .119 | .119 |
| Vander Ploeg (2013)  Canada ([75](#_ENREF_75)) | N = 717, 10.9 ± 0.4 | CS | Pedometers (9 days) | ACTs [parent report]. | Parent encouragement B = 632 (108-1155), *p* < .05 | .073 |
|  |  |  |  |  | Parent modelling B = 890 (67- 1712), *p* < .05 | .073 |
| Voorhees (2005)  USA ([76](#_ENREF_76)) | Not reported | CS | PAQ-C | Asks several questions regarding co-participation and modelling [child report]. | Co-participation 6^th^ grade: Change in predicted PA 0.033 (0.009, 0.048), 95% CI, *p* = .004.  (not enough data for meta-analysis) | N/A |
|  |  |  |  |  | Co-participation 8^th^ grade:  Change in predicted A 0.045 (0.022, 0.069), 95% CI, p < .001.  (not enough data for meta-analysis) | N/A |
| Wenthe (2009)  USA ([77](#_ENREF_77)) | N = 103, 13 ± 0.3 years | CS | PAQ-A and accelerometers (up to 5 days) (total MVPA) | Sallis scale for family and friend support [child report]. | Friend support on % MVPA *r* = .16 | .16 |
|  |  |  |  |  | Family support (% MVPA) β = .32 | .32 |
| Williams (2010)  USA ([78](#_ENREF_78)) | N = 96, 13-19 years | CS | GLTEQ | Social Influences Scale [child report]. | Family support total PA (METS) *r* = .238 | .238 |
|  |  |  |  |  | Friend support total PA (METS) *r* = .261 | .261 |
|  |  |  |  |  | Total support PA (METS) β = .144 | .144 |
| Williams (2011)  Australia ([79](#_ENREF_79)) | N = 184, 15.1 ± 1.2 years | CS | APARQ | Sallis scale (2002) with slight adaptations and modelling with single-item [parent report]. | Parent modelling OR 0.53 (0.22-1.27) 95% CI | -.172 |
|  |  |  |  |  | Parent support OR 5.36 (1.76-16.34), 95% CI | .42 |
| Wilson (2009)  Australia ([80](#_ENREF_80)) | N = 113, 13.9 ± 0.6 years | CS | 3DPAR | No information on where scale derived from. Measures father, mother, best friend and teacher role modelling, co-participation, encouragement and instrumental support [child report]. | Anglo-Australian average daily METS father instrumental support β = .38 | .38 |
|  |  |  |  |  | Vietnamese-Australian average daily METs father co-participation β = -.23  (not enough studies for meta-analysis) | N/A |
|  |  |  |  |  | Vietnamese-Australian on average daily METs teacher instrumental support β = .32  (not enough studies for meta-analysis) | N/A |
|  |  |  |  |  | Vietnamese-Australian on average daily METs mother co-participation β = .43; Vietnamese-Australian on MVPA mother co-participation β = .40; Anglo-Australian on VPA mother co-participation β = -.34. Mean = .163 | .163 |
|  |  |  |  |  | Anglo-Australian on MVPA mother encourage β = .33 | .33 |
|  |  |  |  |  | Anglo-Australian on VPA teacher encourage β = .31  (not enough studies for meta-analysis) | N/A |
|  |  |  |  |  | Anglo-Australian on VPA mother instrumental support β = .43 | .43 |
| Wu (2003)  Taiwan ([81](#_ENREF_81)) | N = 383, 13.5 ± 0.7 years | CS | CAAL | Social support and modelling scales (Garcia et al, 1995) [child report]. | Friend support β = .26 | .26 |
|  |  |  |  |  | Parent support β = -.08 | -.08 |
| Young (2014)  USA ([82](#_ENREF_82)) | N = 4461, 6^th^, 8^th^, and 11^th^ grade | CS | Accelerometers (7 days) | Sallis scale and social network scale, custom scales for teacher and boy support [child report]. | Parameter (SE), *p* value |  |
|  |  |  |  |  | 6^th^ grade friend support 0.35 (0.13), *p* < .01 | .065 |
|  |  |  |  |  | 8^th^ grade friend support 0.19 (0.09), *p* = .04 | .037 |
|  |  |  |  |  | 8^th^ grade (2) friend support 0.32 (0.18), *p* = .08 | .065 |
|  |  |  |  |  | 11^th^ grade friend support 0.28(0.23), *p* = .22 | .051 |
|  |  |  |  |  | 6^th^ grade family support 0.03 (0.09) *p* = .70 | .01 |
|  |  |  |  |  | 8^th^ grade family support -0.01 (0.06), *p* = .84 | -.004 |
|  |  |  |  |  | 8^th^ grade (2) family support -0.08 (0.11), *p* = .08 | -.028 |
|  |  |  |  |  | 11^th^ grade family support 0.07(0.13), *p* = .61 | .021 |
|  |  |  |  |  | 6^th^ grade teacher support -0.2(0.14), *p* = .16 | -.035 |
|  |  |  |  |  | 8^th^ grade teacher support -0.16(0.1), *p* = .09 | -.031 |
|  |  |  |  |  | 8^th^ grade (2) teacher support -0.19(0.2), *p* = .35 | -.035 |
|  |  |  |  |  | 11^th^ grade teacher support -0.11(0.24), *p* = .65 | -.019 |
|  |  |  |  |  | 6^th^ grade boy support -0.15(0.10), *p* = .14  (not enough studies for meta-analysis) | N/A |
|  |  |  |  |  | 8^th^ grade boy support 0.02 (0.07), *p* = .76  (not enough studies for meta-analysis) | N/A |
|  |  |  |  |  | 8^th^ grade (2) boy support -0.13 (0.15), *p* = .39  (not enough studies for meta-analysis) | N/A |
|  |  |  |  |  | 11^th^ grade boy support 0.38 (0.19), *p* = .04  (not enough studies for meta-analysis) | N/A |
|  |  |  |  |  | 11^th^ grade friend modelling 0.45 (0.26), *p* = .09 | .07 |
| Zakarian (1994)  USA ([83](#_ENREF_83)) | N = 815, 15.88 ± 1.17 | CS | Subjective – single item question for VPA and activity checklist (for vigorous PA outside school) | Social support: Friend, family, teacher and coach. Modelling: Family, friend, teacher and coach [child report]. Unclear where scales derived from. | Family support β = .14 | .14 |
|  |  |  |  |  | Coach support β = .06  (not enough studies for meta-analysis) | N/A |
|  |  |  |  |  | Family modelling β = .05 | .05 |
|  |  |  |  |  | Coach modelling β = .04  (not enough studies for meta-analysis) | N/A |
|  |  |  |  |  | Friend support β = -.03 | -.03 |
|  |  |  |  |  | Teacher modelling β = -.03  (not enough studies for meta-analysis) | N/A |
|  |  |  |  |  | Teacher support β = .01 | .01 |
|  |  |  |  |  | Friend modelling β = .01 | .01 |
| Zook (2014)  USA ([84](#_ENREF_84)) | N = 589, 12.29 ± 1.48 years | Longitudinal | Objective - Accelerometers (7 days) | Modified Sallis scale and social network instrument [child report]. | OR (95%CI) |  |
|  |  |  |  |  | Friend support 1.19 (1.04-1.35) | .048 |
|  |  |  |  |  | Family support 1.11 (1.02-1.20) | .029 |
|  |  |  |  |  | Friend modelling 1.22 (0.78-1.89) | .055 |
|  |  |  |  |  | Friend co-participation 1.34 (1.13-1.59) | .080 |

**Physical activity measures:** CLASS-C The Children’s Leisure Activities Study Survey Questionnaire – Chinese version**;** APARQ – Adolescent Physical Activity Recall Questionnaire; 3DPAR - 3-day physical activity recall questionnaire**;** 7DPAR – 7-day Physical Activity Recall Questionnaire**;** SOPLAY – System for Observing Play and Leisure Activity in Youth (objective observations)**;** LTEQ – Leisure Time Exercise Questionnaire; CPA – The Children’s Physical Activity scale; PAI – Physical Activity Interview**;** SAPAC – Self Administered Physical Activity Checklist**;** COPA – Calcium, Osteoporosis and Physical Activity Survey**;** CAAL – The Child/Adolescent Activity Log**;** GLTEQ – Godin’s Leisure Time Exercise Questionnaire.

**Social support measures:** ACTs – Activity Support Scale; FSS – Family Support Scale (Felton et al, 2002)**;** SIS – Social Influences Scale (Saunders et al., 1997); CPAC – The Children’s Physical Activity Correlates questionnaire.

**References**

1. Anderssen N, Wold B. Parental and peer influences on leisure-time physical activity in young adolescents. Res Q Exerc Sport. 1992;63(4):341-8.

2. Baskin ML, Thind H, Affuso O, Gary LC, LaGory M, Hwang SS. Predictors of moderate-to-vigorous physical activity (MVPA) in African American young adolescents. Annals of behavioral medicine : a publication of the Society of Behavioral Medicine. 2013;45 Suppl 1:S142-50.

3. Bauer KW, Nelson MC, Boutelle KN, Neumark-Sztainer D. Parental influences on adolescents' physical activity and sedentary behavior: longitudinal findings from Project EAT-II. Int J Behav Nutr Phys Act. 2008;5:12.

4. Bauer KW, Neumark-Sztainer D, Fulkerson JA, Hannan PJ, Story M. Familial correlates of adolescent girls' physical activity, television use, dietary intake, weight, and body composition. International Journal of Behavioral Nutrition & Physical Activity. 2011;8:25.

5. Beets MW, Pitetti KH, Forlaw L. The Role of Self-efficacy and Referent Specific Social Support in Promoting Rural Adolescent Girls' Physical Activity. American Journal of Health Behavior. 2007;31(3):227-37.

6. Beets MW, Vogel R, Chapman S, Pitetti KH, Cardinal BJ. Parent's social support for children's outdoor physical activity: Do weekdays and weekends matter? Sex Roles. 2007;56(1-2):125-31.

7. Bradley R, McRitchie S, Houts R, Nader P, O'Brien M, Network tNECCR. Parenting and the decline of physical activity from age 9 to 15. International Journal of Behavioral Nutrition and Physical Activity. 2011;8(1):33.

8. Brown BA, Frankel BG, Fennell M. Hugs or Shrugs: Parental and Peer Influence on Continuity of Involvement in Sport by Female Adolescents. Sex Roles. 1989;20(7/8):397-412.

9. Bungum TJ, Vincent ML. Determinants of physical activity among female adolescents. American Journal of Preventive Medicine. 1997;13(2):115-22.

10. Burns C, Murphy JJ, MacDonncha C. Year in school and physical activity stage of change as discriminators of variation in the physical activity correlate profile of adolescent females. Journal of Physical Activity and Health. 2014;11 (4):721-8.

11. Canfield JA. Models of physical activity and sedentary behavior [doctoral dissertation]2012.

12. Cheng LA, Mendonca G, de Farias JC. Physical activity in adolescents: analysis of the social influence of parents and friends. J Pediatr (Rio J). 2014;90(1):35-41.

13. Crawford D, Cleland V, Timperio A, Salmon J, Andrianopoulos N, Roberts R, et al. The longitudinal influence of home and neighbourhood environments on children's body mass index and physical activity over 5 years: the CLAN study. International journal of obesity (2005). 2010;34(7):1177-87.

14. Crimi K, Hensley LD, Finn KJ. Psychosocial correlates of physical activity in children and adolescents in a rural community setting. International Journal of Exercise Science. 2009;2(4):230-42.

15. Davison KK. Activity-related support from parents, peers, and siblings and adolescents' physical activity: Are there gender differences? . Journal of Physical Activity & Health. 2004;1(4):363-76.

16. Davison KK, Downs DS, Birch LL. Pathways linking perceived athletic competence and parental support at age 9 years to girls' physical activity at age 11 years. Research Quarterly for Exercise and Sport. 2006;77(1):23-31.

17. de Farias JC, Reis RS, Hallal PC. Physical activity, psychosocial and perceived environmental factors in adolescents from Northeast Brazil. Cadernos de Saude Publica. 2014;30(5):941-51.

18. Deflandre A, Lorant J, Gavarry O, Falgairette G. Physical activity and sport involvement in French high school students. Perceptual & Motor Skills. 2001;92(1):107-20.

19. Dewar DL, Plotnikoff RC, Morgan PJ. Testing social-cognitive theory to explain physical activity change in adolescent girls from low-income communities. Research Quarterly for Exercise and Sport. 2013;84(4):483-91.

20. DiLorenzo TM, Stucky-Ropp RC, Vander Wal JS, Gotham HJ. Determinants of exercise among children. II. A longitudinal analysis. Preventive Medicine. 1998;27(3):470-7.

21. Dishman RK, Dunn AL, Sallis JF, Vandenberg RJ, Pratt CA. Social-cognitive correlates of physical activity in a multi-ethnic cohort of middle-school girls: two-year prospective study. Journal of Pediatric Psychology. 2010;35(2):188-98.

22. Dishman RK, Saunders RP, Motl RW, Dowda M, Pate RR. Self-efficacy moderates the relation between declines in physical activity and perceived social support in high school girls. Journal of Pediatric Psychology. 2009;34(4):441-51.

23. Dollman J, Lewis NR. Interactions of socioeconomic position with psychosocial and environmental correlates of children's physical activity: An observational study of South Australian families. International Journal of Behavioral Nutrition and Physical Activity. 2009;6(56).

24. Dowda M, Dishman RK, Pfeiffer KA, Pate RR. Family support for physical activity in girls from 8th to 12th grade in South Carolina. Preventive Medicine. 2007;44(2):153-9.

25. Duncan SC, Duncan TE, Strycker LA, Chaumeton NR. A Cohort-Sequential Latent Growth Model of Physical Activity From Ages 12 to 17 Years. Annals of Behavioral Medicine. 2007;33(1):80-9.

26. Edwardson CL, Gorely T, Pearson N, Atkin A. Sources of activity-related social support and adolescents' objectively measured after-school and weekend physical activity: Gender and age differences. Journal of Physical Activity and Health. 2013;10 (8):1153-8.

27. Eime RM, Harvey JT, Craike MJ, Symons CM, Payne WR. Family support and ease of access link socio-economic status and sports club membership in adolescent girls: a mediation study. International Journal of Behavioral Nutrition and Physical Activity. 2013;10(50).

28. Frenn M, Malin S, Villarruel AM, Slaikeu K, McCarthy S, Freeman J, et al. Determinants of physical activity and low-fat diet among low income African American and Hispanic middle school students. Public Health Nurs. 2005;22(2):89-97.

29. Graham DJ, Bauer KW, Friend S, Barr-Anderson DJ, Nuemark-Sztainer D. Personal, behavioral, and socioenvironmental correlates of physical activity among adolescent girls: Cross-sectional and longitudinal associations. Journal of Physical Activity and Health. 2014;11(1):51-61.

30. Graham DJ, Wall MM, Larson N, Neumark-Sztainer D. Multicontextual correlates of adolescent leisure-time physical activity. American Journal of Preventive Medicine. 2014;46(6):605-16.

31. Gregson JF, Colley A. Concomitants of sport participation in male and female adolescents. International Journal of Sport Psychology. 1986;17(1):10-22.

32. He L, Ishii K, Shibata A, Adachi M, Nonoue K, Oka K. Mediation effects of social support on relationships of perceived environment and self-efficacy with school-based physical activity: a structural equation model tailored for Japanese adolescent girls. Open Journal of Preventive Medicine. 2013;3(1):42-50.

33. Huang WY, Wong SH, Salmon J. Correlates of physical activity and screen-based behaviors in Chinese children. Journal of Science and Medicine in Sport. 2013;16(6):509-14.

34. Jackson L, Cumming SP, Drenowatz C, Standage M, Sherar LB, Malina RM. Biological maturation and physical activity in adolescent British females: The roles of physical self-concept and perceived parental support. Psychology of Sport and Exercise. 2013;14(4):447-54.

35. Jago R, Davison KK, Brockman R, Page AS, Thompson JL, Fox KR. Parenting styles, parenting practices, and physical activity in 10- to 11-year olds. Preventive Medicine. 2011;52(1):44-7.

36. Jago R, Wood L, Sebire SJ, Edwards MJ, Davies B, Banfield K, et al. School travel mode, parenting practices and physical activity among UK year 5 and 6 children. BMC Public Health. 2014;14(370).

37. Kahn JA, Huang B, Gillman MW, Field AE, Austin SB, Colditz GA, et al. Patterns and determinants of physical activity in U.S. adolescents. Journal of Adolescent Health. 2008;42(4):369-77.

38. Kelly EB, Parra-Medina D, Pfeiffer KA, Dowda M, Conway TL, Webber LS, et al. Correlates of physical activity in black, Hispanic, and white middle school girls. J Phys Act Health. 2010;7(2):184-93.

39. Keresztes N, Piko BF, Pluhar ZF, Page RM. Social influences in sports activity among adolescents. Journal of The Royal Society for the Promotion of Health. 2008;128 (1):21-5.

40. Kirby J, Levin KA, Inchley J. Parental and peer influences on physical activity among Scottish adolescents: A longitudinal study Journal of Physical Activity and Health. 2011;8(6):785-93.

41. Kitzman-Ulrich H, Wilson DK, Van Horn ML, Lawman HG. Relationship of body mass index and psychosocial factors on physical activity in underserved adolescent boys and girls. Health Psychology. 2010;29(5):506-13.

42. Krishnamoorthy JS. The transmission of physical activity and related cognitions among African-American adolescent females and their primary female caregiver [doctoral dissertation]2002.

43. Kuo J, Young DR, Voorhees CC, Haythornthwaite JA. Associations Between Family Support, Family Intimacy, and Neighborhood Violence and Physical Activity in Urban Adolescent Girls. American Journal of Public Health. 2007;97(1):101-3.

44. Kurc AR, Leatherdale ST. The effect of social support and school- and community-based sports on youth physical activity. Can J Public Health. 2009;100(1):60-4.

45. Lee KS, Loprinzi PD, Trost SG. Determinants of physical activity in Singaporean adolescents. International Journal of Behavioral Medicine. 2010;17(4):279-86.

46. Leggett C, Irwin M, Griffith J, Xue L, Fradette K. Factors associated with physical activity among Canadian high school students. Int J Public Health. 2012;57(2):315-24.

47. Lenhart CM, Patterson F, Brown MD, O'Brien MJ, Nelson DB. Disparity in Physical Activity Among Urban Youth: An Ecologically Guided Assessment. American Journal of Health Education. 2014;45(4):219-28.

48. Leslie E, Kremer P, Toumbourou JW, Williams JW. Gender differences in personal, social and environmental influences on active travel to and from school for Australian adolescents. . Journal of Science and Medicine in Sport. 2010;13(6):597-601.

49. Ling J, Robbins LB, Resnicow K, Bakhoya M. Social support and peer norms scales for physical activity in adolescents. American Journal of Health Behavior. 2014;38(6):881-9.

50. Lubans DR, Morgan PJ. Social, psychological and behavioural correlates of pedometer step counts in a sample of Australian adolescents. Journal of Science and Medicine in Sport. 2009;12(1):141-7.

51. McGuire MT, Hannan PJ, Neumark-Sztainer D, Cossrow NHF, Story M. Parental correlates of physical activity in a racially/ethnically diverse adolescent sample. Journal of Adolescent Health. 2002;30(4,Suppl):253-61.

52. Morgan CF, McKenzie TL, Sallis JF, Broyles SL, Zive MM, Nader PR. Personal, social, and environmental correlates of physical activity in a bi-ethnic sample of adolescents. Pediatric Exercise Science. 2003;15(3):288-301.

53. Morrissey JL, Wenthe PJ, Letuchy EM, Levy SM, Janz KF. Specific types of family support and adolescent non-school physical activity levels. Pediatr Exerc Sci. 2012;24(3):333-46.

54. Motl RW, Dishman RK, Saunders RP, Dowda M, Pate RR. Perceptions of physical and social environment variables and self-efficacy as correlates of self-reported physical activity among adolescent girls. J Pediatr Psychol. 2007;32(1):6-12.

55. O'Loughlin J, Paradis G, Kishchuk N, Barnett T, Renaud L. Prevalence and correlates of physical activity behaviors among elementary schoolchildren in multiethnic, low income, inner-city neighborhoods in Montreal, Canada. Ann Epidemiol. 1999;9(7):397-407.

56. Patnode CD, Lytle LA, Erickson DJ, Sirard JR, Barr-Anderson D, Story M. The relative influence of demographic, individual, social, and environmental factors on physical activity among boys and girls. International Journal of Behavioral Nutrition and Physical Activity. 2010;7:79.

57. Pearson N, Timperio A, Salmon J, Crawford D, Biddle SJH. Family influences on children's physical activity and fruit and vegetable consumption. International Journal of Behavioral Nutrition and Physical Activity. 2009;6.

58. Pis MB. Physical activity social support, self-efficacy, and self-definition in adolescents: a correlational cross-sectional comparative study [doctoral dissertation]2006.

59. Price SM, McDivitt J, Weber D, Wolff LS, Massett HA, Fulton JE. Correlates of Weight-Bearing Physical Activity Among Adolescent Girls: Results From a National Survey of Girls and Their Parents. Journal of Physical Activity & Health. 2008;5(1):132-45.

60. Ramanathan S, Crocker PRE. Correlates of physical activity among adolescent youth in India. International Journal of Sport Psychology. 2013;44(2):111-27.

61. Raudsepp L, Viira R. Influence of parents' and siblings' physical activity on activity levels of adolescents. European Journal of Physical Education. 2000;5(2):169-78.

62. Raudsepp L. The relationship between socio-economic status, parental support and adolescent physical activity. Acta Paediatr. 2006;95(1):93-8.

63. Raudsepp L, Viira R. Changes in physical activity in adolescent girls: a latent growth modelling approach. Acta Paediatr. 2008;97(5):647-52.

64. Reynolds KD, Killen JD, Bryson SW, Maron DJ, Taylor CB, Maccoby N, et al. Psychosocial predictors of physical activity in adolescents. Preventive Medicine. 1990;19(5):541-51.

65. Sallis JF, Prochaska JJ, Taylor WC, Hill JO, Geraci JC. Correlates of physical activity in a national sample of girls and boys in Grades 4 through 12. Health Psychology. 1999;18(4):410-5.

66. Sallis JF, Taylor WC, Dowda M, Freedson PS, Pate RR. Correlates of vigorous physical activity for children in grades 1 through 12: Comparing parent-reported and objectively measured physical activity. . Pediatric Exercise Science. 2002;14:30-44.

67. Saunders RP, Motl RW, Dowda M, Dishman RK, Pate RR. Comparison of Social Variables for Understanding Physical Activity in Adolescent Girls. American Journal of Health Behavior. 2004;28(5):426-36.

68. Sawka KJ, McCormack GR, Nettel-Aguirre A, Blackstaffe A, Perry R, Hawe P. Associations between aspects of friendship networks, physical activity, and sedentary behaviour among adolescents. Journal of Obesity. 2014;2014(632689).

69. Schofield L, Mummery WK, Schofield G, Hopkins W. The association of objectively determined physical activity behavior among adolescent female friends. Res Q Exerc Sport. 2007;78(2):9-15.

70. Shokrvash B, Majlessi F, Montazeri A, Nedjat S, Rahimi A, Djazayeri A, et al. Correlates of physical activity in adolescence: a study from a developing country. Global Health Action. 2013;6:6.

71. Shafer AB. Psychosocial Determinants of Physical Activity in a Sample of Undergraduate College Students. 2012;Ph.D.:119.

72. Sharma SV, Hoelscher DM, Kelder SH, Diamond PM, Day RS, Hergenroeder AC. A path analysis to identify the psychosocial factors influencing physical activity and bone health in middle-school girls. Journal of Physical Activity & Health. 2009;6(5):606-16.

73. Taymoori P, Rhodes RE, Berry TR. Application of a social cognitive model in explaining physical activity in Iranian female adolescents. Health Education Research. 2010;25(2):257-67.

74. Thompson WM, Berry D, Hu J. A church-based intervention to change attitudes about physical activity among black adolescent girls: a feasibility study. Public Health Nursing. 2013;30(3):221-30.

75. Ploeg KAV, Kuhle S, Maximova K, McGavock J, Wu B, Veugelers PJ. The importance of parental beliefs and support for pedometer-measured physical activity on school days and weekend days among Canadian children. BMC Public Health. 2013;13.

76. Voorhees CC, Murray D, Welk G, Birnbaum A, Ribisl KM, Johnson CC, et al. The Role of Peer Social Network Factors and Physical Activity in Adolescent Girls. American Journal of Health Behavior. 2005;29(2):183-90.

77. Wenthe PJ, Janz KF, Levy SM. Gender similarities and differences in factors associated with adolescent moderate-vigorous physical activity. Pediatric Exercise Science. 2009;21(3):291-304.

78. Williams RA. Psychosocial factors and physical activity among black adolescent females. 2010;Ed.D.:110.

79. Williams SL, Mummery WK. Links between adolescent physical activity, body mass index, and adolescent and parent characteristics. Health Education & Behavior. 2011;38(5):510-20.

80. Wilson AN, Dollman J. Social influences on physical activity in Anglo-Australian and Vietnamese-Australian adolescent females in a single sex school. Journal of Science and Medicine in Sport. 2009;12(1):119-22.

81. Wu S-Y, Pender N, Noureddine S. Gender differences in the psychosocial and cognitive correlates of physical activity among Taiwanese adolescents: a structural equation modeling approach. International Journal of Behavioral Medicine. 2003;10(2):93-105.

82. Young D, Saksvig BI, Wu TT, Zook K, Xia L, Champaloux S, et al. Multilevel Correlates of Physical Activity for Early, Mid, and Late Adolescent Girls. Journal of Physical Activity & Health. 2014;11(5):950-60.

83. Zakarian JM, Hovell MF, Hofstetter CR, Sallis JF, Keating KJ. Correlates of vigorous exercise in a predominantly low SES and minority high school population. Preventive Medicine. 1994;23(3):314-21.

84. Zook KR, Saksvig BI, Wu TT, Young DR. Physical activity trajectories and multilevel factors among adolescent girls. Journal of Adolescent Health. 2014;54(1):74-80.
